# Effects of sensory room intervention on autonomic function in healthy adults: A pilot randomized controlled trial

**DOI:** 10.1371/journal.pone.0319649

**Published:** 2025-04-23

**Authors:** Hikari Otsuka, Keisuke Irie, Tomohiro Kogata, Azumi Onitsuka, Hiroyuki Inadomi

**Affiliations:** 1 Department of Advanced Occupational Therapy, Human Health Sciences, Graduate School of Medicine, Kyoto University, Kyoto, Japan; 2 Japan Society for the Promotion of Science, Tokyo, Japan; Virginia Tech: Virginia Polytechnic Institute and State University, UNITED STATES OF AMERICA

## Abstract

Sensory rooms are those equipped with various visual, auditory, and other sensory items that can be adjusted according to user preferences. Although several studies have reported the effectiveness of sensory rooms, their physiological effects remain unclear. This pilot study aims to investigate the effect of sensory rooms on vagal function, mood states, and attentional functions. Thirty-nine healthy young adults were randomly divided into the sensory room intervention (SRI) and sedentary activity (SA) groups, and given a 30-minute intervention. The SRI group spent time in a dimly lit room with beaded cushions and aroma oils. The SA group engaged in activities such as handicrafts and puzzles. We compared changes in respiratory sinus arrhythmia (RSA) at rest, RSA variability during discomfort sensory stimulation, mood states, and attentional functions between the groups, both before and after the intervention. As a result, 1) SRI significantly increased RSA compared with SA. 2) It also reduced the variability of RSA in response to specific sensory stimuli compared with SA. 3) However, no significant differences existed in negative mood or attentional function between the groups. The results suggest that sensory rooms might contribute to the sensory modulation, including that of the autonomic nervous system. Further investigation with larger samples in clinical settings, particularly among individuals with sensory modulation issues and mental illness, is necessary to confirm and generalize these findings.

## 1. Introduction

In acute psychiatric units, the use of seclusion and restraints, which can cause harmful physical or psychological effects on inpatients, remains a global challenge [1]. In recent years, sensory rooms have gained attention as alternatives to these restrictive interventions, promoting personal calm through sensory modulation [2–5].

The sensory rooms are equipped with various visual, auditory, olfactory, and tactile sensory goods that can be adjusted according to the user’s preferences [6]. Sensory room specifications vary depending on the target population and objective of the intervention. When targeting people with severe developmental disabilities or dementia, the sensory rooms are called snoezelen rooms or multisensory environments, and encourage relatively strong sensory stimulation and communication with the user (e.g., seeing twinkling lights and jumping into a ball pool) [7,8]. Conversely, the sensory rooms used for relaxation and calming in mental health settings are called sensory modulation rooms, and are installed in inpatient psychiatric wards [3,6,9]. The effects of sensory rooms in psychiatry include relaxation of the body and mind; calmness; reduction of stress, anxiety, and distress levels; improved sense of well-being, self-esteem, and self-control; and reduced physical restraint and medication use [3,5,10–12]. This study focuses on the effects of sensory rooms primarily used in psychiatry for relaxation purposes.

Respiratory sinus arrhythmia (RSA) is a physiological indicator associated with sensory modulation [13]. The high-frequency heart rate variability (HRV) is referred to as RSA [14], and its amplitude reflects the degree to which the vagus nerve, part of the parasympathetic nervous system, inhibits the heartbeat [15,16].

The two vagal functions are: vagal tone, which describes the activity of the vagus nerve at rest; and vagal reactivity, which describes the magnitude of the response to external stimuli [16,17]. People with with atypical sensory modulation (e.g., hypersensitivity) have low RSA at rest (i.e., vagal tone) and increased RSA (i.e., vagal reactivity) when exposed to sensory stimuli [18–20]. Therefore, the RSA at rest and during sensory stimulation may be an appropriate indicator of the sensory modulation.

As mentioned above, several studies have reported the effectiveness of sensory rooms, including their calming and relaxing effects and their role in reducing stress and anxiety [3,5,10–12]. However, many reports are at risk of bias, including confounding factors and participant selection, and the physiological effects of sensory rooms remain largely unknown [3,21]. Moreover, the HRV, as the primary outcome, may be affected by medications such as selective serotonin reuptake inhibitors [22], complicating the assessment of the pure effects of sensory rooms in psychiatric patients taking such medications. Additionally, activities used in conventional psychiatric occupational therapy affect mood states as well as the autonomic nervous system [23,24], although the difference in effects between these activities and sensory-based interventions remains unclear.

In order to examine these aspects, we conducted a preliminary investigation into how sensory room intervention (SRI) affects vagal function by randomly dividing healthy adults into two groups, measuring the RSA, and comparing its effect with that of sedentary activity (SA). Particularly, we focused on the resting vagal tone and variability of vagal activity when participants were exposed to sensory stimuli. We also investigated whether mood state and attention function improve as secondary outcomes.

We tested three hypotheses:

1) The SRI increases vagal tone compared to SA.
2) SRI suppresses fluctuations of vagal reactivity during external sensory stimulation compared to SA.
3) SRI decrease negative mood and improve attention function compared to SA.

## 2. Methods

### 2.1 Study design and procedure

This study was designed as a two-arm randomized controlled trial, dividing participants into SRI and SA groups. For participant assignment, we employed a stratified block randomization method, using sex as the stratification variable. The random allocation sequence was generated using the RAND function in Microsoft Excel. Participants were university students recruited from the author’s institution through the university website. The enrolment period was from March 9 to June 28, 2023, and the follow-up period was until August 2, 2023. A single author, H.O., was responsible for generating the random assignment sequence, enrolling participants, and assigning them to intervention groups, following the procedure described above. Another author, K.I., quantified the RSA, including visual noise processing, with the groups blinded.

**Fig 1:**
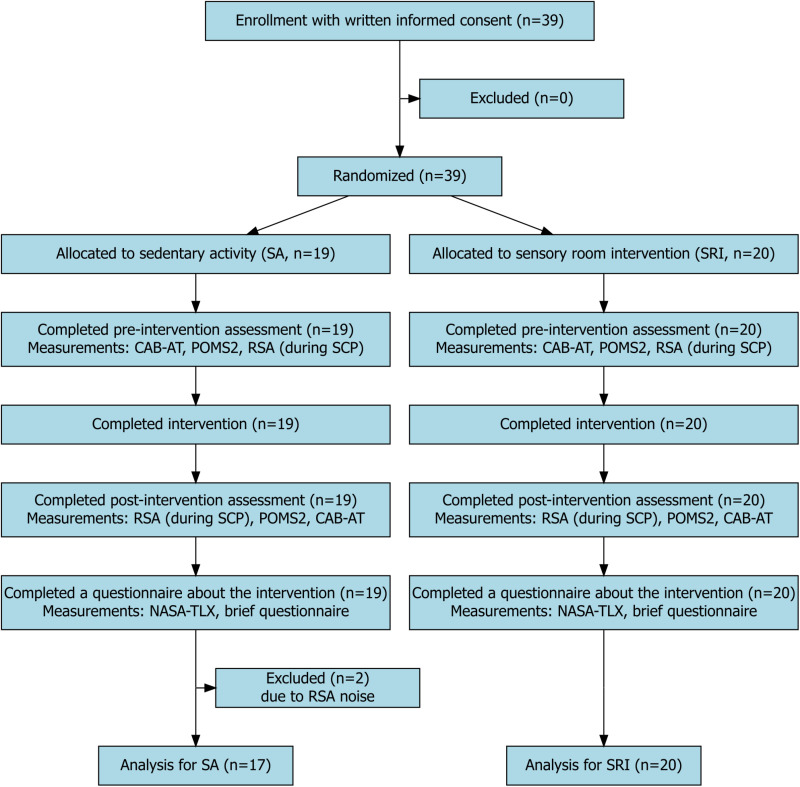
Experimental Procedure Flow Diagram. Note. CAB-AT, Concentration Cognitive Assessment; POMS2, Profile of Mood States 2nd Edition; RSA, Respiratory sinus arrhythmia; SCP, Sensory Challenge Protocol; NASA-TLX, Task Load Index.

A flowchart of the protocol is presented in Fig 1. All participants were screened to determine whether they met the eligibility criteria and provided demographic information using the Japanese Adult Reading Test, Autism Spectrum Quotient, Schizotypal Personality Questionnaire, and Adolescent/Adult Sensory Profile. Both prior to and after the intervention, we conducted several assessments, which have been described below. Participants were informed of their group assignment after completing the pre-intervention assessment. After all the evaluations, the participants completed a questionnaire regarding each intervention.

### 2.2 Participants

Thirty-nine healthy young adults participated in this study. The inclusion criteria were as follows: being over 18 years of age and having a Japanese Sensory Inventory mini score ≥  1. The exclusion criteria were as follows: history of heart disease; organic or functional impairment of vision, hearing, touch, taste, or smell; and history of mental, developmental, language comprehension, or motor dysfunction. Two participants were excluded from the analysis because they exhibited pulse-wave noise in more than half of the time series. Therefore, 37 participants were included in the analysis.

We ran a sensitivity analysis on the analysis of variance model equivalent to the linear mixed model reported below, testing the 2 interventions ×  2 pre–post effects on resting RSA (corresponding to Hypothesis 1). This analysis revealed that the sample size allowed for the detection of the smallest population effect size of 1.91 (Cohen’s f), interpreted as a large effect. This means that our study was able to detect a large effect of the intervention on the resting RSA.

### 2.3 Interventions

For the SRI and SA groups, all pre-post testing and interventions were conducted in a quiet private room measuring approximately 3m ×  3m. The intervention time for both groups was 30 minutes. During the intervention, the tester remained in the same room, but did not converse with the participants and recorded the items and participants’ activities.

#### 2.3.1 *Sensory room intervention.*

**Fig 2:**
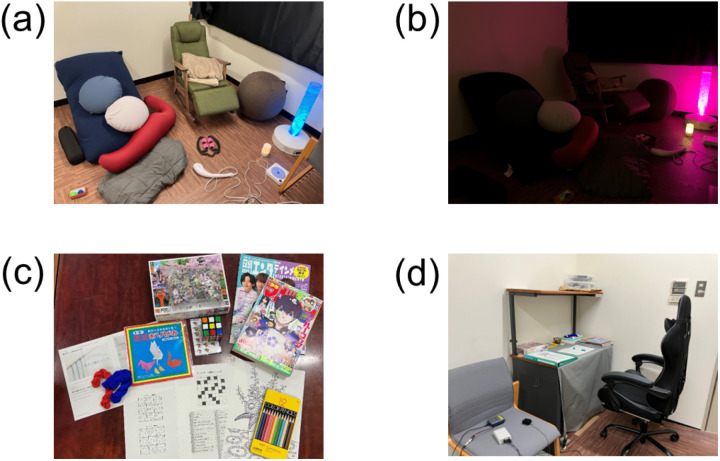
Setting of the sensory room. (a) Sensory room items and (b) dimly lit setting with only a mini-bubble tube. (c) Sedentary activity items and (d) the setting for sedentary activity tasks and Sensory Challenge Protocol.

Based on previous studies [12,25–27], we designed SRI environments by incorporating the following items: life-size beaded cushion, rocking chair, mini bubble tube, two small beaded cushions, balance ball, stretch pole, hugging pillow, weighted blanket, light blanket, two squeezes, electric hand massager, foot roller, music player with healing music, aroma diffuser, and various aroma oils (orange, lemon, lavender, eucalyptus, and tea tree) (Fig 2, S1 Appendix). To ensure the effective selection of items, authors H.O., K.I. and H.I selected the set based on previous research as well as their own expertise. They also visited hospitals that use sensory rooms to calm patients with mental disorders and received practical advice from local therapists, which informed the SRI design. The examiner briefed participants on how to use each item and encouraged them to create a comfortable and relaxed environment. Initially, the participants selected one aroma oil and then chose the preferred items and postures for the start of the intervention. Subsequently, the mini-bubble tube (featuring changing lights to create a calming sensory experience), healing music, and aroma diffuser remained active, leaving it to the participants’ choice to turn them off if desired. During the intervention, they were allowed to adjust their postures and change items as desired. Additionally, they were reminded not to fall asleep during the intervention. The room was kept either dark or dimly lit (Fig 2).

#### 2.3.2 *Sedentary activity.*

Based on conventional psychiatric occupational therapy activities, we designed the SA to include activities with fewer than three Metabolic Equivalents. Participants freely chose their activities from among coloring books, origami, crosswords, sudoku, jigsaw puzzles, Rubik’s cubes, macramé, and reading magazines or comic books (Fig 2). They engaged in these activities in a well-lit room in a seated position, and were allowed to change their activities during the intervention. All sedentary activities were designed to exclude explicit sensory input and did not use the same objects as the SRI group.

### 2.4 Measures

In both groups, we compared several key metrics before and after the intervention: RSA values, RSA response to discomforting sensory stimuli, Profile of Mood States 2nd Edition (POMS2) scores, and Concentration Cognitive Assessment (CAB-AT) scores. The POMS2 scores and CAB-AT, which are secondary outcomes, were assessed before and after the RSA measurements. Following the completion of all assessments, the participants completed a Task Load Index (NASA-TLX) to report their mental workload and a brief questionnaire regarding the intervention experience.

#### 2.4.1 *Measurement of RSA during the sensory challenge protocol.*

We used the Sensory Challenge Protocol (SCP) as a protocol involving multiple sensory stimuli. The SCP is a stimulus-presenting task that involves auditory, visual, olfactory, tactile, and vestibular sensory stimuli [18–20,28]. The task began with a rest period (3 minutes), followed by six sensory stimuli (tone, visual, siren, olfactory, tactile, and vestibular), a recovery period (3 minutes), and a prolonged auditory stimulus (2 minutes). Before the SCP began, participants were informed of each stimulus and asked to look at the fixation point on the display. S2 Appendix provides the details of each sensory stimulus. Additionally, subjective discomfort levels were measured to examine changes in emotions in response to sensory stimuli caused by the intervention. Participants responded to the subjective discomfort level of each SCP modality on an 11-point Likert scale, with scores ranging from 0 (not at all uncomfortable) to 10 (very uncomfortable).

#### 2.4.2 *RSA calculation.*

A portable pulse-wave measuring device, Polypul II (Nihonsanteku, Osaka, Japan), was used to measure the photoplethysmogram waveform (sampling rate: 1000 Hz) and the RSA was calculated based on the pulse waveform. The RSA (units =  ln[ms^2^]) was noise processed using CardioEdit (Brain-Body Center, University of Illinois, Chicago, USA), and quantified using CardioBatch (Brain-Body Center, University of Illinois, Chicago, USA), which implements the Poges-Borer method [15,16,29]. Our previous study details noise processing and quantification [19].

We calculated the RSA values for each of the nine stages of the SCP: rest period, six types of sensory stimulation (sound, sight, siren, smell, touch, and vestibular), recovery, and prolonged auditory stimulation.

#### 2.4.3 *Assessment of mood state and attentional function.*

The POMS2 is a standardized self-administered questionnaire that assesses mood states. We used the Japanese version of the all-item version for adults (65 items). Participants answered questions about their current mood. Total mood disturbance (TMD) is calculated as a T-score, with higher scores indicating more negative mood [30].

The CAB-AT is a computer-based assessment of cognitive function, with sufficient reliability and validity [31]. The total attention score, which is the percentile score normalized by age and sex, was calculated to be in the range of 0–800 points. Higher scores indicated better attentional function.

#### 2.4.4 *Mental workload and impressions of each intervention.*

The Japanese version of the NASA-TLX was administered to ascertain whether the effects in the two intervention groups were due to differences in the task load required for the activities. Participants responded to six items on a 20-point scale, which included questions on mental and physical demands. The overall task load index was calculated on a scale of 0–100. A higher index indicated greater mental load [32].

We created a brief questionnaire to ascertain whether the effects of the two groups’ interventions were due to differences in their activity preferences. Using a Likert scale ranging from 1 (not at all disagree) to 7 (quite agree), participants responded to four questions about SRI goods and SA activities, related to their satisfaction with goods/activities, preference for goods/activities, interest in goods/activities, and absence of goods/activities that they would like to use.

This study was approved by the Kyoto University Graduate School and Faculty of Medicine Ethics Committee (Approval No. C1604-1). Written informed consent was obtained from all the participants. At the time the study was initiated, the authors were unfortunately not aware of the International Committee of Medical Journal Editors policy requiring prospective registration of all interventional clinical trials. Upon becoming aware of this policy, we promptly registered this trial with the Japan Registry of Clinical Trials (Registration number: jRCT1052240104). The authors confirm that all ongoing and related trials for this intervention are registered. This study protocol is available as supporting information.

### 2.5 Statistical analysis

We adopted a Bayesian framework to address the uncertainty inherent in the main outcomes (RSA, POMS2, and CAB-AT). In this approach, uncertainty is represented by probability distributions, reflecting the range of possible parameter values based on both data and prior information. This allowed us to test hypotheses using criteria such as posterior probabilities and credible intervals, rather than relying on p-values, which provide only point estimates of significance without considering uncertainty.

A Bayesian generalized linear mixed model was used to analyze the RSA values. The model was designed to assess three independent variables: type of treatment (SRI or SA), time point (pre or post), SCP condition, as well as their interactions. Moreover, the random effects for individual participants were considered in the model. The model formula is as follows:


$$\text{RSA}∼\text{Treatment×Time×Condition}+\;\left(\text{1} |\text{ID}\right)\text{.}$$

For the post hoc analysis, we also explored the contrast between each variable to assess the extent to which each intervention could modulate RSA.

Similarly, a Bayesian generalized linear mixed model was used to assess the effects of the interventions on POMS2 and CAB-AT scores. The model considered the following parameters: treatment type, time point, interaction, and random effects for individual participants. The formula for this analysis was:


$$\text{value}∼\text{Time×Treatment}+\;\left(\text{1} |\text{ID}\right)$$

The computation was performed using Markov Chain Monte Carlo (MCMC) sampling. A weakly informative Gaussian distribution was used in the analysis. To confirm the convergence of MCMC sampling, convergence diagnostics were verified, including Rhat values (Gelman-Rubin diagnostic) below 1.01 and an effective sample size (ESS) greater than 1000. We calculated the Bayes Factor (BF) to quantify how well our hypothesis-based model fit the observed data compared with a model that considers only random effects. A BF value greater than 1.0 suggests that the model based on our hypothesis provides relatively stronger evidence to explain the observed data.

In this study, we report the 95% credible interval (CI) determined from the highest density interval (HDI) of the posterior distribution, indicating the range in which the actual parameter is likely to fall with 95% probability. The HDI corresponds to a range of posterior distributions in which all points within the interval have a higher probability density than those outside of the interval. Additionally, two key indicators, Probability of Direction (PD) and Region of Practical Equivalence (ROPE), were used to comprehensively understand the statistical results beyond mere significance. The PD quantifies the probability of parameter consistency, offering insights into effect trends (e.g., whether the parameter is negative or positive). The ROPE identifies a range where the effects are considered negligible, thereby aiding in evaluating practical significance. A PD threshold of 0.975 (indicating a high probability for the direction of the effect) was applied, and the ROPE range was set as a negligible effect size according to Cohen’s d, with a ROPE percentage under 2.5% suggesting “probably significant” and under 1.0% suggesting “significant” [33].

As additional survey items, the results of the SCP subjective discomfort levels and impressions of the intervention (NASA-TLX and a brief questionnaire) were also analyzed. After confirming the normality of the data using the Shapiro-Wilk test, a Wilcoxon signed-rank test with Bonferroni correction was performed. The significance level of 5% was Bonferroni-corrected separately for each SCP domain and each item in the questionnaire on impressions of the intervention.

All analyses were performed using R 4.3.2, brms (version 2.20.4) [34], BayesFactor (version 0.9.12.4.7), and BayestestR (version 0.13.1) [35] packages. Detailed codes for these analyses is provided in S3 Appendix.

## 3. Results

Demographic data are displayed in Table 1.

**Table 1:** Participant demographics.

Variable	SA, n = 17^1^	SRI, n = 20^1^
Age (year)	21.8 (2.1)	21.9 (4.5)
Sex (F/M)	8/9	10/10
Education (year)	15.0 (2.0)	14.5 (1.9)
IQ	113.4 (5.1)	113.8 (6.9)
AQ	24.1 (7.9)	20.5 (7.7)
SPQ	25.1 (11.7)	20.1 (10.3)
AASP		
Low Registration	33.3 (10.1)	32.4 (9.4)
Sensation Seeking	38.2 (9.7)	39.3 (7.0)
Sensory Sensitivity	39.3 (10.5)	35.1 (8.4)
Sensation Avoiding	39.1 (8.4)	35.6 (8.9)
Temperature (Celsius)	25.6 (1.7)	24.9 (1.9)
Humidity (relative humidity, %)	62.2 (16.6)	66.1 (14.6)

^1^Mean (SD); n.

Note. SA, Sedentary activity; SRI, Sensory room intervention; IQ, Intelligence Quotient; AQ, Autism spectrum quotient; SPQ, Schizotypal Personality Questionnaire; AASP, Adolescent/Adult Sensory Profile. IQ was assessed using the Japanese Adult Reading Test.

### 3.1 Estimated effect on RSA

**Fig 3:**
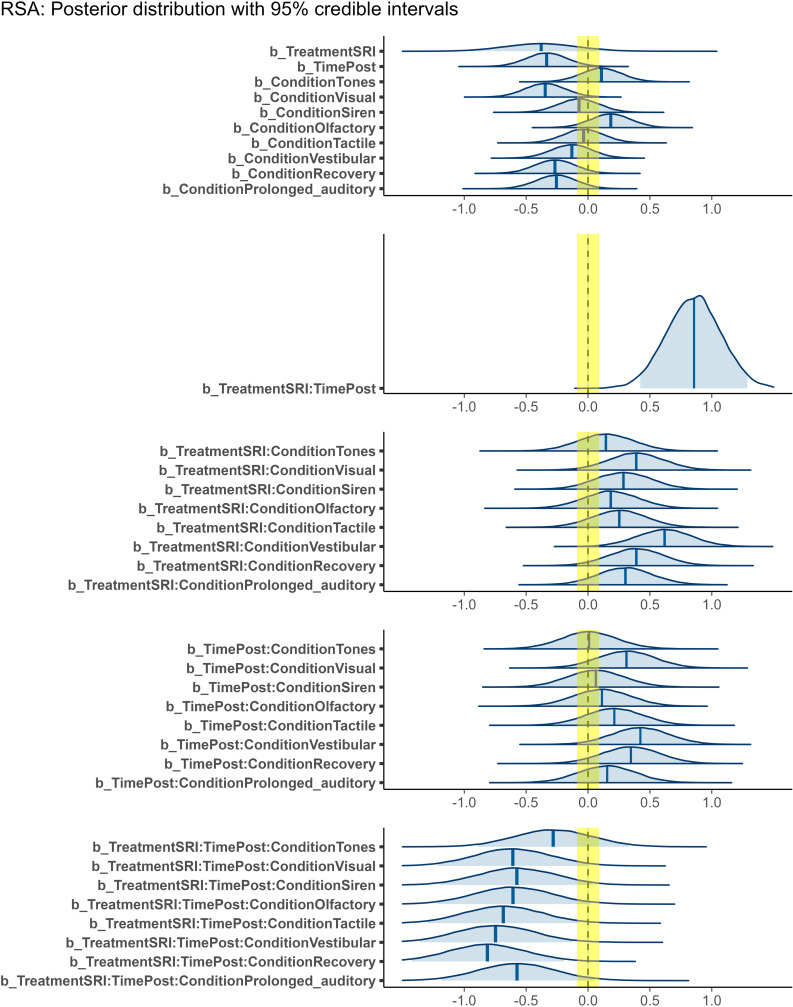
Posterior distribution for RSA. Note. SRI, Sensory room intervention. These parameters pertain to a Bayesian generalized linear mixed model involving three factors: Treatment (comprising two levels: SA and SRI), Time (comprising two levels: Pre and Post), and Condition (comprising nine levels: Resting, Tones, Visual, Siren, Olfactory, Tactile, Vestibular, Recovery, and Prolonged auditory). Single-factor terms represent main effects, whereas combinations of factors represent interaction effects. The regression coefficients (indicated by “b_”) represent the estimated effects for each variable or interaction. Shaded areas of the distributions (light blue) indicate the 95% credible interval (CI) from the lower limit to the upper limit. The yellow range indicates region of practical equivalence (ROPE) range. The bias from 0.0 with respect to each distribution density represents the impact on respiratory sinus arrhythmia (RSA), indicating a positive/negative effect.

**Fig 4:**
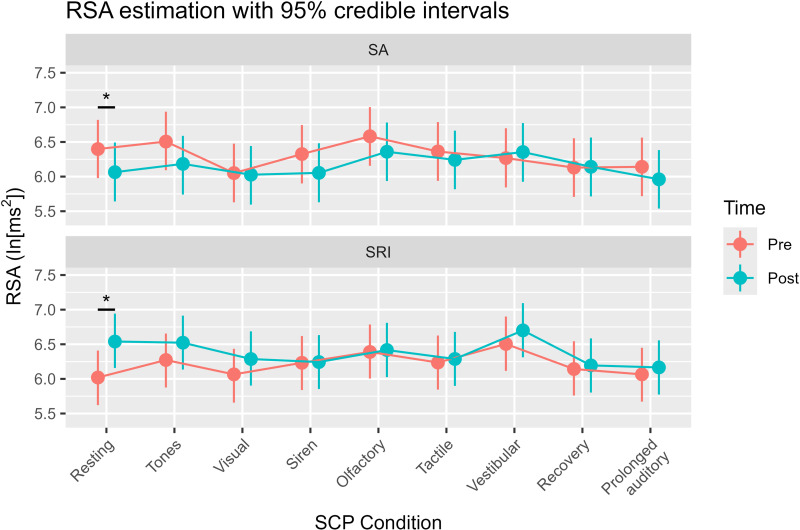
Estimating RSA Variability in Each SCP Condition. Note. RSA, Respiratory sinus arrhythmia; SA, Sedentary activity; SRI, Sensory room intervention; SCP, Sensory Challenge Protocol. * : PD >  0.975. Median (points) and 95% confidence intervals (bars) for RSA estimates. In the Resting condition, the increase or decrease in RSA was opposite between SRI and SA.

Table 2 shows the model summary of the RSA variability (see also Fig 3 regarding posterior distributions). S2 Table presents the mean RSA during the SCP, and S1 Fig shows the RSA variability during the SCP in both groups. Our hypothesis-based model demonstrated robust performance compared to the null model (BF =  27.6). We found a significant interaction between time and treatment (median =  0.86, PD =  1.00, CI [0.43, 1.29], ROPE% =  0.0), indicating that SRI was more effective in increasing the RSA than SA. Particularly, we identified opposite effects of RSA in resting conditions between SRI and SA (Fig 4). When comparing the RSA between pre- and post-intervention as a post hoc analysis, SRI significantly increased RSA [Δdifference =  0.52, CI [0.23, 0.82], PD =  0.999, ROPE% =  0.0], whereas SA tended to decrease RSA [Δdifference =  −0.33, CI [−0.65, −0.02], PD =  0.981, ROPE% =  5.3]. RSA reflects vagal tone activity; therefore, these findings illustrate that SRI effectively increases vagal tone.

**Table 2:** Estimation of RSA model parameters during the SCP.

Parameter			Estimate β (median)	95% CI (lower, upper)	PD	ROPE percentage	Rhat	ESS
Intercept			6.40	5.98, 6.82	**1.000**	**0.0**	1.00	10509.4
Variables								
**Treatment**	**Time**	**Condition**						
SRI	–	–	−0.38	−0.95, 0.19	0.901	10.5	1.00	10097.3
–	Post	–	−0.33	−0.65, −0.02	**0.981**	4.1	1.00	11816.8
–	–	Tones	0.11	−0.21, 0.43	0.749	34.4	1.00	17043.1
–	–	Visual	−0.35	−0.67, −0.03	**0.982**	3.2	1.00	16173.8
–	–	Siren	−0.07	−0.39, 0.25	0.673	38.3	1.00	16787.9
–	–	Olfactory	0.18	−0.14, 0.50	0.871	23.5	1.00	18099.3
–	–	Tactile	−0.04	−0.36, 0.28	0.586	40.9	1.00	17172.2
–	–	Vestibular	−0.13	−0.45, 0.19	0.789	31.5	1.00	17668.2
–	–	Recovery	−0.27	−0.59, 0.05	0.950	11.4	1.00	16750.7
–	–	Prolonged auditory	−0.26	−0.58, 0.05	0.942	12.9	1.00	16660.0
SRI	Post	–	0.86	0.43, 1.29	**1.000**	**0.0**	1.00	11551.1
SRI	–	Tones	0.14	−0.29, 0.57	0.743	25.9	1.00	17037.1
SRI	–	Visual	0.39	−0.05, 0.82	0.961	6.3	1.00	17411.2
SRI	–	Siren	0.29	−0.16, 0.71	0.901	14.5	1.00	16369.1
SRI	–	Olfactory	0.18	−0.24, 0.63	0.799	22.4	1.00	17416.0
SRI	–	Tactile	0.25	−0.20, 0.67	0.871	16.9	1.00	17208.5
SRI	–	Vestibular	0.62	0.17, 1.04	**0.997**	**0.0**	1.00	17007.1
SRI	–	Recovery	0.39	−0.05, 0.82	0.960	6.3	1.00	16577.0
SRI	–	Prolonged auditory	0.30	−0.15, 0.72	0.914	13.0	1.00	16692.8
–	Post	Tones	0.01	−0.44, 0.46	0.516	31.1	1.00	16763.4
–	Post	Visual	0.31	−0.14, 0.75	0.910	13.1	1.00	15457.6
–	Post	Siren	0.06	−0.40, 0.51	0.608	29.5	1.00	16464.7
–	Post	Olfactory	0.11	−0.33, 0.57	0.685	27.0	1.00	17624.9
–	Post	Tactile	0.21	−0.23, 0.66	0.824	20.2	1.00	16580.7
–	Post	Vestibular	0.42	−0.02, 0.88	0.967	4.8	1.00	16794.3
–	Post	Recovery	0.35	−0.11, 0.80	0.931	10.5	1.00	16578.2
–	Post	Prolonged auditory	0.16	−0.30, 0.60	0.747	24.4	1.00	16280.3
SRI	Post	Tones	−0.28	−0.89, 0.34	0.814	15.2	1.00	16126.2
SRI	Post	Visual	−0.61	−1.20, 0.02	0.974	2.4	1.00	16734.9
SRI	Post	Siren	−0.58	−1.17, 0.05	0.968	3.6	1.00	15874.7
SRI	Post	Olfactory	−0.61	−1.22, 0.01	0.971	2.6	1.00	17018.8
SRI	Post	Tactile	−0.68	−1.30, −0.08	**0.986**	**0.2**	1.00	16152.7
SRI	Post	Vestibular	−0.75	−1.37, −0.13	**0.991**	**0.0**	1.00	16369.4
SRI	Post	Recovery	−0.81	−1.43, −0.20	**0.995**	**0.0**	1.00	16261.0
SRI	Post	Prolonged auditory	−0.57	−1.19, 0.04	0.965	3.7	1.00	16505.4

Note. These parameters pertain to a Bayesian generalized linear mixed model involving three factors: Treatment (comprising two levels: SA and SRI), Time (comprising two levels: Pre and Post), and Condition (comprising nine levels: Resting, Tones, Visual, Siren, Olfactory, Tactile, Vestibular, Recovery, and Prolonged auditory). Single-factor terms represent main effects, whereas combinations of factors represent interaction effects. Median probability density, 95% credible interval (CI), probability of direction (PD), and percentage in region of practical equivalence (ROPE) are shown. The ROPE range was set from −0.09 to 0.09. PD is 0.975, indicating a high probability for the direction of the effect, and a ROPE percentage under 2.5% suggests “probably significant,” whereas under 1.0% suggests “significant.”

RSA. Respiratory sinus arrhythmia; SCP, Sensory challenge protocol; SA, Sedentary activity; SRI, Sensory room intervention; ESS, Effective sample size.

**Fig 5:**
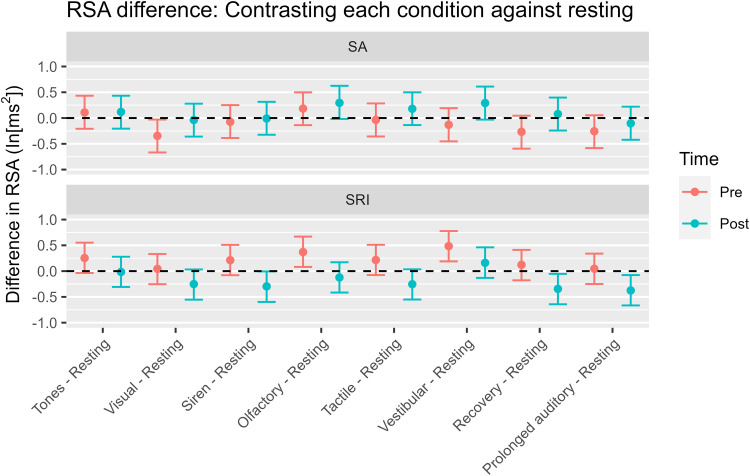
Estimating RSA Variability across Treatment, Time, and Condition. Note. RSA, Respiratory sinus arrhythmia; SA, Sedentary activity; SRI, Sensory room intervention. These values refer to the RSA difference between each condition and the resting state; a negative/positive value indicates that the RSA in the condition decreased/increased compared to the resting state. In all SRI conditions, the post intervention RSA differences were lower than pre intervention, indicating that the SRI intervention induced resilience to stress.

We also found some significant interactions between time, treatment, and stimuli conditions for RSA variability during the SCP (Figs 3, 5 and Table 2). For vestibular stimulation and recovery, we found a significant interaction between time and treatment and a highly significant negative effect (vestibular: PD =  0.991, ROPE% =  0.0; recovery: PD =  0.995, ROPE% =  0.0). Furthermore, when considering PD, we found a significant interaction and negative effect on tactile perception (PD =  0.986, ROPE% =  0.2). The following items were negatively correlated with PD: visual (PD =  0.974), siren (PD =  0.968), olfactory (PD =  0.971), and prolonged auditory (PD =  0.965). Tone showed low significance, but a possible negative effect (PD =  0.814). These findings suggest that SRI is effective in decreasing the RSA during each stimulus of the SCP from the resting RSA compared to SA.

The results of the SCP subjective discomfort levels are shown in Table 3.

**Table 3:** Pre- vs. post-intervention comparison in subjective discomfort levels of the SCP.

Variable	SA, n = 17^1^			SRI, n = 20^1^		
	Pre	Post	p-value^2^	Pre	Post	p-value^2^
Subjective discomfort level						
Tones	7.1 (1.9)	6.8 (1.1)	0.20	6.3 (2.2)	6.1 (2.1)	0.65
Visual	4.0 (2.2)	3.9 (2.1)	0.87	4.3 (1.9)	3.2 (2.1)	0.07
Siren	7.1 (1.9)	7.2 (1.8)	0.90	6.5 (2.2)	6.7 (2.3)	0.72
Olfactory	2.4 (1.7)	2.4 (1.6)	0.99	2.2 (2.4)	1.9 (1.9)	0.71
Tactile	3.0 (2.4)	2.4 (2.0)	0.42	2.6 (2.0)	2.1 (1.7)	0.45
Vestibular	2.7 (2.1)	2.9 (2.3)	0.85	2.0 (1.4)	1.6 (1.1)	0.44
Prolonged auditory	6.6 (1.9)	6.2 (2.1)	0.89	6.9 (2.3)	6.2 (2.2)	0.32

^1^Mean (SD)

^2^Wilcoxon rank sum exact test.

Note. p <  0.007 was considered statistically significant after applying Bonferroni correction. SCP, Sensory challenge protocol; SA, Sedentary activity; SRI, Sensory room intervention.

### 3.2 Estimated effect on POMS2 and CAB-AT

A summary of the POMS2 and CAB-AT models is provided in Table 4. S2 Table, presents the mean POMS2 and CAB-AT scores pre- and post-intervention. Our hypothesis-based model demonstrated robust performance compared to the null model (BF for POMS2 =  986996.4, BF for CAB-AT =  442268609.0). No significant interaction was observed between time and treatment for either item. However, regardless of the group, after the intervention, the TMD of POMS2 decreased significantly (PD =  0.999, ROPE% =  0.0), and the total CAB-AT increased significantly (PD =  0.998, ROPE% =  0.0).

**Table 4:** Estimation of POMS2 and CAB-AT model parameters.

Parameter		Estimate β (median)	95% CI (lower, upper)	PD	ROPE percentage	Rhat	ESS
TMD of POMS2							
Intercept		46.90	43.22, 50.74	**1.000**	**0.0**	1.00	24728.5
Variables							
**Treatment**	**Time**						
SRI	–	−1.92	−7.02, 3.25	0.775	20.0	1.00	24317.1
–	Post	−6.75	−10.74, −2.41	**0.999**	**0.0**	1.00	34557.4
SRI	Post	0.72	−5.02, 6.32	0.599	23.0	1.00	32821.1
Total score of CAB-AT							
Intercept		605.16	559.52, 651.96	**1.000**	**0.0**	1.00	18821.5
Variables							
**Treatment**	**Time**						
SRI	–	−6.27	−69.09, 58.14	0.577	24.9	1.00	17835.0
–	Post	61.37	21.18, 102.08	**0.998**	**0.0**	1.00	45233.1
SRI	Post	−5.73	−62.44, 48.18	0.581	28.4	1.00	42904.5

Note. These parameters pertain to a Bayesian generalized linear mixed model involving two factors: Treatment (comprising two levels: SA and SRI) and Time (comprising two levels: Pre and Post). Single-factor terms represent main effects, whereas combinations of factors represent interaction effects. Median of probability density, 95% credible interval (CI), probability of direction (PD), and percentage in region of practical equivalence (ROPE) are shown. The ROPE range was set from −0.82 to 0.82 for Total mood disturbance (TMD) of Profile of Mood States 2nd Edition (POMS2), −9.82 to 9.82 for Total of Concentration Cognitive Assessment (CAB-AT). SA, Sedentary activity; SRI, Sensory room intervention; ESS, Effective sample size.

### 3.3 Details and impressions of spending time during the intervention

The results of the NASA-TLX, a brief questionnaire, and details of how the participants spent their time during the intervention are presented in Table 5, S3 Table, and S4 Table. NASA-TLX was significantly lower in the SRI group than in the SA group. However, no significant differences existed in satisfaction, preference, interest, or impression of wanting to use between the groups.

**Table 5:** Results of a brief questionnaire and NASA-TLX for each intervention.

Variable	SA, n = 17^1^	SRI, n = 20^1^	p-value^2^
NASA TLX	54.5 (14.6)	19.2 (15.1)	**<0.001**
Subjective scaling			
Satisfaction	6.1 (1.1)	6.5 (0.6)	0.22
Preference	6.4 (0.9)	6.3 (0.7)	0.48
Interest	6.3 (0.9)	6.4 (0.8)	0.84
Impression	1.7 (1.5)	2.0 (1.6)	0.58

^1^Mean (SD),

^2^Wilcoxon rank sum test.

Note. p <  0.01 was considered statistically significant after applying Bonferroni correction. SA, Sedentary activity; SRI, Sensory room intervention; NASA-TLX, Task Load Index.

## 4. Discussion

This pilot study investigated the effects of the sensory room on vagal function, mood states, and attentional functions. Our findings revealed that: 1) SRI notably increased RSA, indicating an increase in vagal tone, compared to SA; 2) when compared to the RSA reactivity from the resting state to specific disturbing stimuli, SRI showed less variability in RSA (i.e., vagal reactivity) than SA; and 3) there was no significant difference in negative mood and attentional performance between the two groups. These findings contribute to our understanding of the mechanisms underlying the effects of sensory rooms on sensory modulation, including the autonomic nervous system.

### 4.1 Vagal tone change

The RSA increase induced by SRI seems to reflect the physiological changes induced by relaxation. Previous studies have reported a close relationship between HRV and emotional state [36,37]. The vagal tone reflects anxiety and relaxation [38]. Our findings indicate that the therapeutic use of a sensory room has a relaxing effect, consistent with those of several previous studies [3,5,12,39]. Previous research has reported that sedative music [40], aromatherapy [41,42], and exposure to colored lights [43] increase parasympathetic activity. Therefore, the effect of each sensory stimulus could have influenced the increase in vagal tone.

### 4.2 Vagal reactivity change

In response to disturbing stimuli, the vagal reactivity post-SRI showed a greater decrease in RSA reactivity compared to SA (especially tactile and vestibular), indicating a more unperturbed reaction to disruptive triggers.

This decrease in vagal reactivity after SRI suggests an alteration in the processing of sensory stimuli. Increased vagal reactivity during stress stimulation reflects self-regulatory efforts or recovery from stress [44]. Therefore, the suppression of the increase in RSA from resting to SCP stimuli may reflect the reduced need for self-adjustment and recovery. Sensory room interventions could affect one aspect of sensory modulation [45], including autonomic nervous system responses; however, further investigation is required to substantiate this.

The novelty of this study is its examination of the effects of the sensory room by focusing on vagal reactivity during disturbing stimuli. People with atypical sensory modulation (e.g., hypersensitivity) have increased vagal reactivity during sensory stimulation [18–20]. However, the adequate suppression of vagal reactivity during stress loading is associated with improved cognitive performance [46]. Therefore, SRI might help people with sensory modulation difficulties to maintain their cognitive performance when they encounter disturbing stimuli in their daily lives.

Many people with mental illnesses exhibit atypical sensory modulation [21,47]. Therefore, SRI may also benefit people with both psychiatric disorders and sensory modulation problems. Furthermore, the subjective discomfort level of the SCP did not change after the intervention; thus, vagal reactivity may be more sensitive to the effects of sensory-based interventions.

### 4.3 Mood state and attentional function changes

Regarding mood states and attentional function, SRI and SA tended to reduce negative mood and improve attentional performance after the intervention; however, for SRI, immediate autonomic regulation did not improve mood state or attentional performance.

Crafts and other creative activities improve mood states [23], and the use of a sensory room reduces distress and regulates emotions [3,39]. Therefore, in the current study, negative mood was reduced in both groups, which may explain the absence of any significant differences between the groups. Regarding changes in attentional function, previous studies have discussed the relationship between the autonomic nervous system and cognitive performance [48]. Long-term SRI may also influence attentional function by causing positive changes in autonomic function; however, such a report has not yet been published. In the future, it will be necessary to demonstrate the effects of repeated therapeutic SRI using physiological indices.

### 4.4 Mental workload, impressions, and items used during the intervention

The results, including the NASA-TLX and a brief questionnaire, revealed that the interventions were characterized by differences in their effects on vagal function and subjective mental load.

The SRI group participants could choose the manner in which they preferred to spend their time in the sensory room, where the time and available items were controlled. However, we do not yet sufficiently understand the specific amount of time and goods that makes SRI effective. To explore the effective use of SRI in clinical practice, it is necessary to consider whether individuals should choose items based on their personal preferences or whether therapists should recommend them according to their sensory profiles.

### 4.5 Safety and adverse events

In this study, the SRI in was conducted with healthy young adults, and no significant adverse events were observed. All participants completed the intervention safely, without any physical or psychological issues attributable to the intervention.

However, previous studies have reported that the use of sensory rooms can lead to adverse effects in patients with mental disorders. For example, Björkdahl, et al. [26] found that the use of sensory rooms sometimes exacerbated symptoms such as auditory hallucinations, self-harm, panic, claustrophobia, and increased anxiety. Therefore, caution should be exercised when using sensory rooms in clinical settings, particularly for patients with mental health conditions.

Additionally, the use of aromatherapy oils should also be approached with care. Some oils with pharmacological effects (e.g., herbs, orange, eucalyptus, tea tree, lavender) have been shown to not only affect emotions but also impact the respiratory system [49]. Therefore, exercising caution is important when using these oils with patients who have respiratory conditions, ensuring appropriate selection and usage.

Future research should further assess these risks and examine the safety of interventions in different patient populations.

### 4.6 Limitations

This study had some limitations. First, the small sample size makes generalizing the results to larger populations difficult. We used Bayesian statistics with random effects to address the sample size issue by accounting for individual variability. However, small sample sizes might affect the robustness of Bayesian a priori information. Future studies with larger sample sizes are required to confirm these results.

Second, the SCP cannot completely eliminate the carryover effects of previous sensory modality stimuli. We employed the SCP because SRI is an intervention involving multisensory stimuli, and because it is closer to the environment of daily life, where people are constantly exposed to multiple sensory stimuli. To further investigate the effects of SRI, it is necessary to focus on the validation of a single stimulus and provide sufficient intervals between different sensory domains.

Third, regarding the intervention, the sensory stimuli included in the SRI and SA could not be completely controlled. In other words, sensory input is also provided by the SA. This study was conducted immediately after the 30 minute intervention, and the duration of the effect was unknown. Moreover, the extent to which the sensory room in this study met the formal definition of a sensory room, particularly one designed for relaxation purposes, is open to debate. Since no clear standards exist regarding the items and usage of sensory rooms, the authors relied on previous studies to guide the selection of items. Participants were informed that the purpose of the sessions was relaxation, and none engaged in active movement during the sessions. However, the authors did not consult directly with experts in sensory room design or architecture. Therefore, the development of a standardized sensory room setup remains an important challenge for future research.

Finally, some limitations regarding the other evaluation indicators existed. POMS2 and CAB-AT were administered after the SCP, which may have diminished the intervention’s effects. The POMS2 was dependent on individual subjectivity, and the CAB-AT results also included learning effects. As such, future studies should be conducted using simpler tasks in which learning effects are less pronounced.

### 4.7 Application of results

Our preliminary results suggest that the sensory rooms may enhance vagal function and self-regulation in response to sensory stimuli. They offer a noninvasive and safe intervention suitable even for patients requiring isolation or restraint. Moreover, as sensory rooms do not necessitate specialized skills, they can be implemented in hospitals, educational settings, workplaces, and various community environments. This study provides initial evidence supporting the effectiveness of sensory room interventions, with our preliminary findings suggesting that the effects of craft activities in conventional psychiatric occupational therapy on mood states and cognitive functions are comparable to those of sensory room interventions. Therefore, in clinical practice, these activities should be selectively provided or combined based on the therapeutic goals and individual client conditions, ensuring the best possible outcomes for patients.

## 5. Conclusion

This study involved a preliminary investigation into the effect of a sensory room on vagal function, mood states, and attentional functions. The findings suggested that the use of a sensory room may increase vagal function compared to static seated activity. However, we found no significant differences in negative mood or attentional performance between the two interventions. These findings suggest that the sensory rooms might contribute to the sensory modulation, including that of the autonomic nervous system. Although these results are promising, the small sample size of this pilot study necessitates cautious interpretation. Further investigation with larger sample sizes is needed in clinical settings with individuals with sensory modulation problems and mental illness, in order to confirm these findings and enhance their generalizability.

## Supporting information

S1 File CONSORT checklist. (DOCX)

S2 File Protocol (Original). (DOCX)

S3 File Protocol (English). (DOCX)

S1 Table Individual raw data. (DOCX)

S2 Table The mean values before and after the intervention for the two groups. (DOCX)

S3 Table Activities engaged in the Sedentary Activity group (n = 17). Values indicate the number of people using each item for each usage time.
(DOCX)

S4 Table Sensory items used in the Sensory Room Intervention group (n = 20). Values indicate the number of people using each item for each usage time.
(DOCX)

S1 Fig Measured values of RSA in pre- and post-intervention during the SCP. (DOCX)

S1 Appendix Details of the sensory room items. (DOCX)

S2 Appendix Details of the Sensory Challenge Protocol. (DOCX)

S3 Appendix Details of the statistical analysis. (DOCX)
