# Supplementary material for: Effects of sensory room intervention on autonomic function in healthy adults: A pilot randomized controlled trial
Source: PLoS One. 2025 Apr 23;20(4):e0319649. doi: 10.1371/journal.pone.0319649 (PMC12017487; doi:10.1371/journal.pone.0319649)
Supplement: S2 File — (DOCX) [file pone.0319649.s002.docx]

研究実施計画書

2022年11月29日　Ver.1

2022年12月6日　Ver.2

2022年12月23日　Ver.3

2023年2月2日　Ver.4

2023年2月21日　Ver.5

2023年2月28日　Ver.6

1. 研究の名称

　　健常成人の感覚処理特性に対するSensory Roomを用いた介入効果の検証：ランダム化比較試験

2.　研究の背景

近年，統合失調症や自閉スペクトラム症（以下，ASD）などの精神疾患を有する方における社会参加の困難さが問題となっており，その原因の一つには感覚処理の障害があるとされる^1)^．感覚処理障害とは，環境内のあらゆる刺激を視覚，聴覚等の情報として知覚し，脳内で情報を統合して，状況の認識や次の行動に繋げるプロセスのことで，精神疾患を有する方は適切な情報の知覚や統合が難しく，環境刺激への過敏な反応や他者の示す表情などの社会的なサインの誤解およびそれに起因する社会的引きこもりが生じるとされている^2)^．

臨床場面において，欧米諸国では感覚処理障害に着目したSensory Roomを用いた介入が一般的に行われている．Sensory Roomとは，感覚刺激を適量に調節できる部屋のことで，効果として心身のリラクゼーションや感覚入力に関するセルフコントロール能力の獲得^3)^が言われているが，いずれも質的調査による報告が多く，エビデンスの検証は不十分である．

一方，従来の精神科作業療法では，手工芸や読書，パズル，軽体操などの多様な作業活動や運動を用いたプログラムが行われている．作業活動を用いた介入は気分状態や認知機能の改善に一定の効果をもたらすとされる^4)5)^．しかし，いずれも感覚に着目した介入ではなく，作業活動と感覚に着目した介入の効果の違いは明らかでない．

また，神経生理学分野では，感覚処理障害と自律神経機能および脳波との関連が示されている．具体的には，ASD児は感覚刺激に暴露した際に副交感神経機能が低下し自律神経機能のバランスが乱れること^6)^や，統合失調症患者では感覚刺激を入力してから検出された情報に注意を向けるなどの認知過程に障害があることが脳波の事象関連電位（event-related potential：ERP）であるN1, MMN（mismatch negativity）, P300にて示されている^7)^．副交感神経機能について，我々は呼吸性洞性不整脈（respiratory sinus arrhythmia：RSA）に着目した．RSAとは，吸気時に心拍が速くなり呼気時に遅くなるという心拍数の変動のことで，呼吸によって変動する迷走神経活動を反映しており，純粋な副交感神経機能の指標として広く用いられている^8)^．

感覚処理特性の偏りは，精神疾患の診断を受けていない健常者にも存在するとされる．健常者を対象とした調査において，感覚過敏傾向の者はそうでない者に比べて聴覚刺激の大きな周波数変化に対するP2事象関連電位の潜時が有意に長いこと^9)^や，感覚刺激への過敏性や感覚刺激から回避する傾向は，日常生活で感じるストレスの大きさと関連しているという報告^10)^がある．

しかし，精神疾患の診断の有無に関わらず，自律神経機能や脳波などの生理学的指標を用いてSensory Roomの有用性を示した報告は，我々が調べた限りでは見当たらず，Sensory Roomの生体反応レベルでの効果は明らかになっていない．

3.　研究の目的および意義

1. 何を明らかにしようとしているのか

本研究では，健常者を対象に，感覚処理特性に着目したSensory Roomを用いた介入（以下，SRI）の有効性を自律神経指標や脳波といった生体指標や主観的な気分状態および認知機能の変化によって示すことを目的とする．これにより感覚処理特性に着目した介入の有用性を基礎的な調査により明らかにする．

リサーチクエスチョンは，健常成人に対するSensory Roomを用いた介入は静的活動と比べて，副交感神経活動および脳波の振幅を増大させ，気分状態や認知機能を向上させるか，である．

1. 医学的．社会的意義は何か

本研究の独自性は，自律神経機能や脳波といった生理学的指標や認知機能の変化によってSensory Roomの治療的効果を示すところにある．健常者におけるSensory Roomの有用性を客観的指標と認知機能向上によって示すことは，感覚処理障害を抱える割合が高いとされる精神疾患を有する方への新たな治療法の提案に大いに貢献すると考える．将来的には，Sensory Roomの有効性を広く周知することで，病院や教育現場および職場における設置に繋げ，発達障害や精神疾患を有する方および健常者も含めた人々のメンタルヘルスケアや作業能率の向上にも繋がることが期待される．加えて，上記の貢献に伴う作業療法士の役割や作業療法の有用性を明確にすることができると考える．

4.　研究対象者の選定方

1. セッティング

地域在住の健常成人及び大学生・大学院生等を対象に，本研究に関する研究対象者募集の案内掲示およびチラシ配布（資料1）を行い，問い合わせがあった者に対し，本研究内容に関して口頭および書面で説明し，対象者を集める．

1. 適格基準

　　以下の選択基準をすべて満たし，除外基準のいずれにも該当しない者を適格として登録する．

・　選択基準

1. 登録時の年齢が18歳以上65歳以下である．
2. 研究参加について，研究対象者本人から文書による同意が得られている．
3. JSI-mini（Japanese Sensory Inventory mini）（資料2）における総スコアが1点以上

設定根拠について，(a)は本研究の対象者を成人としているためと，高齢による心身機能の低下が結果へ影響することを考慮したためである．(c)は本研究の介入対象である感覚処理特性に関して，日常生活上にて何らかの特徴がある人を対象とするためである．

・　除外基準

以下のいずれかの項目に該当する者を研究対象者として用いない．

1. 心疾患の合併症がある
2. 心臓ペースメーカー使用者
3. てんかんの既往がある
4. 視力（矯正可）が両眼で0.7未満
5. 気導聴力が30㏈以上
6. 触覚の知覚低下がある（セメスワインスタインモノフィラメントの2.83番を感知できない）
7. 顕著な味覚および嗅覚異常の自覚がある
8. その他，本研究の課題遂行に支障をきたすと思われる視力障害や聴力障害，言語理解障害，精神障害，運動機能障害の既往歴がある

設定根拠について，(a)および(b)は自律神経計測，(c)は脳波計測に影響する可能性があるためである．また，（d)～（g）は本研究の評価や介入対象となる感覚器官に機能的異常がある人を除外するためである．

・中止基準

1. 研究対象者が中止を希望したとき
2. その他中止するのが適当と判断されたとき

3） 予定研究対象者数およびその設定根拠

本研究の目標症例数は，介入群29名と統制群29名の計58例とする．

設定根拠について，本研究では，メインアウトカムとして介入前後の副交感神経指標（RSA：respiratory sinus arrhythmia）の変化について，介入群と統制群の2群比較（対応のないt検定，片側検定）を行う．G　Power 3.1.9.2 を用い，RSAを用いた先行研究^11)12)^を参照して効果量を0.7と設定し，αエラー=0.05，βエラー=0.20に設定すると，総サンプルサイズは52名となる．さらに，約10％のドロップアウトが起こる可能性を考えて，約58名の研究対象者が必要となる．58名をランダムにSRI群29名と統制群29名に振り分ける．

ASD児や健常児の副交感神経指標および感覚処理特性を調査した先行研究^6)13)^の対象者数はそれぞれ計50名，83名であったことからも，上記の設定人数は概ね妥当と考える．

5.　研究の方法および研究の科学的合理性の根拠

1） デザイン

□　介入研究

・　試験の相：探索的研究

・　比較の方法：前後比較／並行群間比較

・　介入の割付け方法：無作為化

・　評価者盲検化：統計解析担当の研究者のみを盲検化する.

以上より，本研究はランダム化比較試験とする．

2） 方法

本研究への参加を希望し，研究実施者から口頭及び書面にて十分な説明を受けて参加に同意し，適格基準を満たした者を研究対象者として登録する．研究実施者は，割り付け表を用いて，対象者をランダムにSRI群と統制群に割り振り，介入を実施する．その後，対象者のIDとデータのみを統計解析担当である分担研究者のもとに届け，解析を実施する．このように統計解析担当の研究者のみを盲検化することでバイアスを最小化して結果を導く．

実施の流れを図１に示す．研究は，(a)予備調査（スクリーニング），(b)初期評価，(c)介入直前評価，(d)介入，(e)介入直後評価，(f)最終評価の6つの段階に分けられる．以下，各段階について説明する．これらの調査や評価，および介入は全て京都大学大学院医学研究科人間健康科学系専攻にて実施する．


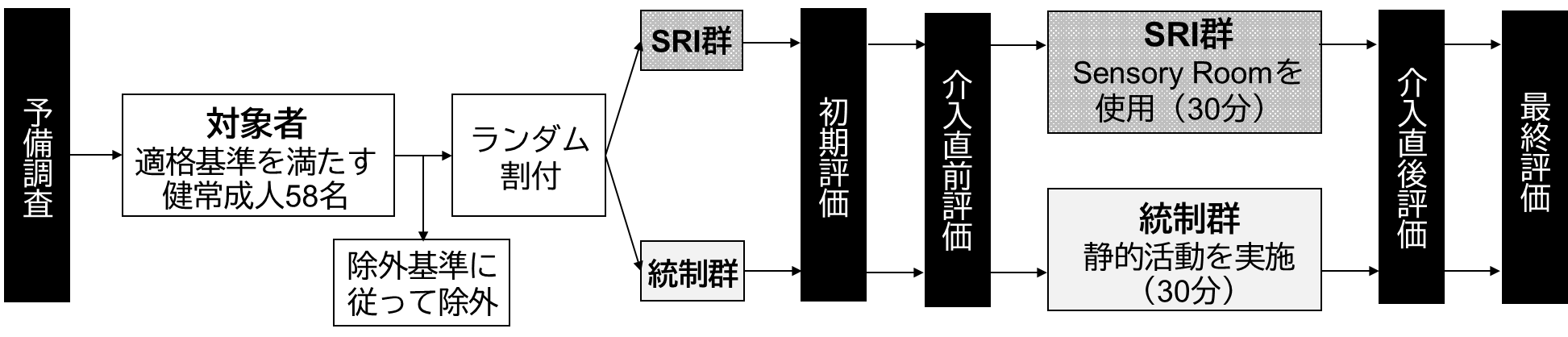


図１：実施の流れ

1. 予備調査（スクリーニング）

主に本研究に適さない研究対象者のスクリーニングの目的と，対象者の精神疾患傾向および感覚処理特性の評価のために行う．測定項目は①対象者の基本属性と②医学的情報，③JSI-mini（Japanese Sensory Inventory mini），④自閉症スペクトラム指数（Autism spectrum Quotient: AQ），⑤統合失調症スペクトラム指数（Schizotypal Personality Questionnaire: SPQ），⑥日本版青年/成人感覚プロファイル（Adolescent/Adult Sensory Profile：A/ASP）の聴取および評価を行う（「3）観察項目とスケジュール」参照）．適否の判断は，適格基準に沿って行い，適格基準を満たさないと判断した場合は，以降の研究には参加をご遠慮いただき，予備調査にかかった時間に応じた謝金を支給して実験終了とする．適格基準を満たした研究対象者を登録後，研究対象者をSRI群と統制群の2群にランダムに割付し，分担研究者（統計解析担当）に対して解析終了まで盲検化する．

1. 初期評価

予備調査に続いて，初期評価に参加いただく．初期評価では①Cognitive Assessment Battery(CAB)を行っていただく．（「3）観察項目とスケジュール」参照）．これにより，介入前の研究対象者の認知機能の経時的評価の基準とする．なお，本評価は，研究対象者の負担軽減と，今後の精神疾患を有する方を対象とした数週間単位の介入研究に向けてのコントロールデータとなる可能性を考慮して，介入の前日に実施する．

1. 介入直前評価

　初期評価の翌日，介入の前に介入直前評価に参加いただく．評価項目は，①Sensory Challenge Protocol（SCP）実施時のRSA計測，②脳波計測，③Profile of Mood States 2nd Edition (POMS2)とする．本研究のメインアウトカムであるRSA計測を最も介入に近い時間帯に実施するため，測定は，POMS2，脳波計測，RSA計測の順に実施する．これらの評価にて，介入前の研究対象者の自律神経機能および脳波の波形の傾向や主観的な気分状態の経時的評価の基準とする．

1. 介入

SRI（Sensory Room Intervention)群はSensory Roomを用いた介入，統制群は静的活動を30分間受けていただく．なお，研究対象者58名のうち同意の得られた約20名に対しては，介入中のリラックスの程度や認知的賦活の程度および睡眠覚醒を捉えるため，脳波および心電図・脈波計測を実施する（資料13）．

≪介入内容≫

1. SRI：約5.0㎡×2.0ｍのSensory Roomを本学の人間健康科学科棟内に作成し，対象者と研究実施者が1人ずつ入室する．なお，入室する研究実施者は作業療法士の免許を取得している者とする．内部には，調光と調色が可能な照明，バブルチューブ，音楽プレーヤーとヒーリングミュージックの音源，加重ブランケット，ビーズクッション，アロマディフューザーとアロマオイル，触覚ボール等を置く．はじめは研究実施者が対象者のA/ASPのスコアに基づいて個人の感覚処理特性に応じた感覚刺激を提案しつつ，徐々に対象者が主体的に感覚刺激を選択および調整できるように促す．研究実施者は対象者の過ごし方を記録する．1回の介入時間はSensory Roomを用いた介入を実施した先行研究を参照し^14)15)^，30分とする．終了時には，Sensory Room内の物品についての感想を簡易アンケートやNASA-TLX (Task Load Index)といった質問紙を用いて聴取する（資料11）．
2. 静的活動：編み物や折り紙等の創作活動や，パズル，読書，動画鑑賞等の椅子座位で行う約3METs未満の低強度な活動（資料12）を実施してもらう．場所は①と同じSensory Room内とするが，①で用いる物品やその他の感覚刺激に意識を向ける物品は使用せず，照明点灯下にて行う．対象者と研究実施者が1人ずつ入室し，対象者には部屋内にある作業活動から自由に選択してもらう．1回の介入はSRIと同様に30分とする．終了時には，活動内容についての感想を簡易アンケートやNASA-TLXといった質問紙を用いて聴取する（資料11）．
3. 介入直後評価

30分間の介入の後，介入直後評価を実施する．評価項目は，介入直前評価と同じ①Sensory Challenge Protocol（SCP）実施時のRSA計測，②脳波計測，③Profile of Mood States 2nd Edition (POMS2)からなる（「3）観察項目とスケジュール」参照）．実施順はRSA計測，脳波計測，POMS2の順に行う．所要時間は60分程度である．介入群と統制群のそれぞれにおいて，介入が研究対象者の自律神経機能および脳波の波形の傾向や主観的な気分状態にどのような変化を与えたかを検討する．

1. 最終評価

介入直後評価の翌日，最終評価を実施する．最終評価は，初期評価で実施した①CABを行う（「3）観察項目とスケジュール」参照）．所要時間は30分程度である．介入群と統制群のそれぞれにおいて，介入が研究対象者の認知機能にどのような変化を与えたかを検討する．

3） 観察・検査・調査・報告項目とスケジュール

- 測定項目，測定方法，測定者または測定機関

下記に測定項目と方法を述べる．測定は全て研究実施者が実施する．

1. 予備調査で実施する項目

以下を聴取，評価する．所要時間は30分である．

1. 対象者の基本属性（年齢，性別，教育歴，職業歴，利き手，知的機能）（資料3）：利き手の評価には，日本語版FLANDERS利き手テスト（資料4）を，知的機能の評価にはJART (Japanese Adult Reading Test)（資料5）を用いる．
2. 医学的情報（既往歴，現病歴，視力，聴力，触覚，味覚，嗅覚）（資料3）：視力の評価には視力検査表国際標準式5m用，聴力はMimi Hearing Test，触覚はセメスワインスタインモノフィラメントを用いる．
3. JSI-mini（Japanese Sensory Inventory mini）（資料2）：JSI-R(Japanese Sensory Inventory Revised)の簡易版で，全20項目の自己記入式である．感覚刺激の受け取り方の傾向を簡易的に把握できる．
4. 自閉症スペクトラム指数（Autism spectrum Quotient: AQ）日本語版（資料6）:健常範囲の知能を持つ成人を対象に個人の自閉症傾向を評価する自己回答形式の質問紙．自閉性障害を特徴づける症状を示す5領域（社会的スキル，注意の切り替え，細部への関心，コミュニケーション，想像力）が10問ずつで構成され，計50項目ある．回答は「あてはまる」「どちらかといえばあてはまる」「どちらかといえばあてはまらない」「あてはまらない」の4つから選択する．合計スコアは0-50点の範囲で，カットオフ値は33点以上である．
5. 統合失調症スペクトラム指数（Schizotypal Personality Questionnaire: SPQ）日本語版（資料7）：健常者の統合失調型パーソナリティの特性を包括的に測定できる自己記入式の質問紙．回答は「はい」「いいえ」の2件法で，全部で74項目ある．
6. 日本版青年/成人感覚プロファイル（Adolescent/Adult Sensory Profile：A/ASP）（資料8）：感覚処理の傾向を「低登録」「感覚探求」「感覚過敏」「感覚回避」で評定する自己記入形式の質問票．対象は11歳から82歳である．各15項目で計60項目あり，4つの各特性のスコアが取りうる値の範囲は15点以上75点以下である．
7. 初期評価および最終評価で実施する項目

以下を評価する．所要時間は30分程度である．

1. Cognitive Assessment Battery(CAB)（資料9）：注意や集中力，知覚，記憶，実行機能，調整力などの様々な認知領域の機能や，身体的，心理的，社会的幸福を測定できる．評価はオンラインで実施する．
2. 介入直前評価および介入直後評価で実施する項目

以下を評価する．所要時間は60分程度である．

1. Sensory Challenge Protocol（SCP）実施時のRSA計測：SCPは，①安静期間（3分），②視覚や聴覚などの6種の感覚刺激が各々提示されるタスク期間，③回復期間（3分），④持続的聴覚刺激（2分）の4段階で構成される．RSAの計測には携帯型脈波測定装置PolyPul（ニホンサンテク）を使用する．
2. 脳波計測：オドボール課題やメニースタンダード課題^16)^で構成されたタスクを提示して脳波を計測する．オドボール課題は高頻度の標準刺激と低頻度の逸脱刺激から成る．課題の聴覚刺激は，周波数，提示確率の順に，標準刺激は1000Hz，80％，逸脱刺激は1100Hz，20％とし，刺激の持続時間は50ms，強度50㏈，刺激時間間隔500ms，逸脱刺激の加算回数が60回となるように作成する．メニースタンダード課題は，オドボール課題よりも聴覚刺激の周波数や持続時間の組み合わせが多く，刺激の出現が予測し難い課題である．脳波計測に用いる機器はPowerLab（バイオリサーチセンター）およびactiCHamp Plus（PHSIO-TECH）を使用し，電極位置は国際10-20法に従い，サンプリング周波数は1000Hzとする．
3. Profile of Mood States 2nd Edition (POMS2) 日本語版　オンライン版（資料10）：怒りや混乱，抑うつなどの気分状態を評価する質問紙．「怒り－敵意」「混乱－当惑」「抑うつ－落込み」「疲労－無気力」「緊張－不安」「活気－活力」「友好」の7つの尺度があり，それらを総合した総合的気分状態（TMD）得点も算出される．持続する感情状態（過去１週間どのように感じたか）と一過性の感情（今現在どのように感じているか）の2種類を評価できるが，本調査では介入直前と介入直後におけるそれぞれの一過性の感情について尋ねる．成人用の全項目版（65項目）を使用する．所要時間は約8～10分である．
4. 介入中に取得する項目

　同意の得られた約20名に対して，介入中に以下の項目を取得する．いずれもサンプリング周波数は1000Hzとする．

1. 脳波計測：32Chの脳波電極から介入中の脳波を測定し，高速フーリエ変換を用いた周波数分析にて各帯域のパワースペクトルを部位ごとに算出する．測定機器はactiCHamp Plus（PHSIO-TECH）を使用し，電極位置は国際10-20法に従う．
2. 心電図・脈波計測：心電図電極は右鎖骨下，左鎖骨下，下胸部の3点誘導とし，得られた波形からRSA値を算出する．測定機器はactiCHamp Plus（PHSIO-TECH）または携帯型脈波測定装置PolyPul（ニホンサンテク）を使用する．

- 測定スケジュール：

実験の流れを図２に示す．評価および介入は，3日間に分けて実施する．1日目は，説明と同意を得たのち，予備調査と初期評価を実施する．2日目は，介入直前評価と介入，介入直後評価を実施し，3日目に最終評価を実施する．それぞれの評価項目は，適宜休憩を挟みながら実施する．


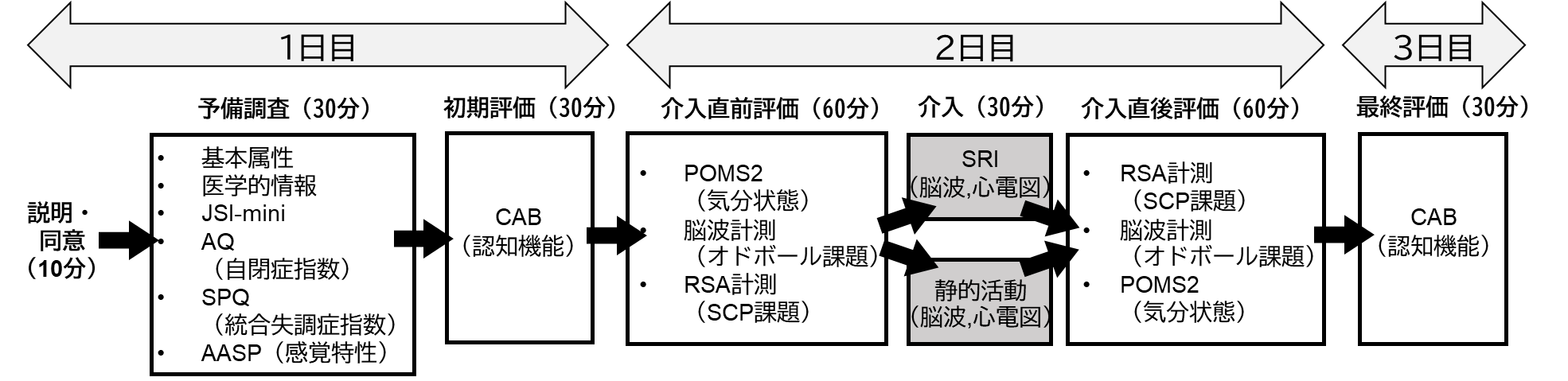


図２：測定スケジュール

4） 解析の概要

- - 主要評価項目

(a)介入前後でのSCPの感覚刺激によるRSA値の変動の差

- - 副次的評価項目

(a)介入前後での脳波振幅の差，(b)介入前後でのPOMS2のスコアの差，(c)初期および最終評価時のCABのスコアの差, (d)AQのスコアとRSA値との相関，（e）SPQのスコアとRSA値との相関，(f)A/ASPのスコアとRSA値との相関，（g）介入中の脳波の帯域ごとのパワースペクトル，（h）介入中のRSA

- - 主な解析方法

統計的有意水準をp＜0.05に設定してデータ解析を行う．また，片側検定とする．

基本属性，医学的情報，Sensory Room使用時の観察記録は記述統計としてまとめる．RSA値はSCPの安静期間と各感覚刺激時の平均値の差を算出し，脳波振幅は提示課題の逸脱刺激波形から標準刺激波形を減算して得られる潜時約200～400msの陽性または陰性成分の振幅を得る．

ベースラインとなる予備調査や初期評価および介入直前評価の調査項目においてSRI群と統制群の間に差がないことを確認する目的で，2群間で対応のないt検定を実施する．次に，両群におけるRSA値，脳波振幅，POMS2のスコアの介入直前評価時と介入直後評価時の差分を対応のないt検定にて群間比較する．また，CABのスコアの初期評価と最終評価時の差分を対応のないt検定にて群間比較する．なお，ベースラインで測定する項目にて2群間に有意差があった場合は，層別解析あるいはサブグループ解析を実施する．

さらに，予備調査のAQやSPQおよびA/ASPスコアとRSA値，脳波振幅，POMS2のスコアの介入直前評価時と介入直後評価時の差分との関連について相関係数を求める．脳血流量は，両群における介入開始時と終了時の各5分間の平均値の差分を求め，対応のないt検定にて群間比較する．

介入中の測定項目については，脳波の帯域ごとのパワースペクトルとRSAについて群間で対応のないt検定を行う．

6.　研究期間

1） 研究対象者登録期間

　研究機関の長の実施許可日より4年間とする．

2） 研究対象者観察期間

　　　研究対象者観察期間は予備調査時から3日間とする．

3） 研究実施期間

研究機関の長の実施許可日から5年間を研究実施期間とする．

7.　インフォームド・コンセント（以下，「IC」）を受ける手順

予備調査の前に研究に関する説明文書を利用し，十分に説明する．測定項目，測定時間，介入内容，侵襲が少ない事等を説明し，その後対象者の自由意思により参加を決定してもらう．参加の同意と署名をもって，インフォームド・コンセント手続きとする．

8.　個人情報等の取扱い

1)　研究で取り扱う資料・情報等の個人情報等の種類

　　本研究は仮名化された情報を取り扱う．

2)　1)の作成の時期と方法

データ等の個人情報は研究責任者のもとに一旦集め，対象者の氏名をID番号に変換することで仮名化を行う．解析担当である分担研究者は氏名がID番号に変換されたデータを用いて解析を行う．氏名とIDを記載した対応表は，対象者のプライバシーに十分配慮しデータが外部に漏出することがないよう鍵のかかる保管庫，もしくは外部とのネットワーク接続がない持ち出し不可能なPCで厳重に管理し，論文公表後10年間は保管することにする．研究結果を学術雑誌や学会などでの発表することもあるが，個人のプライバシーは厳重に守られ，個人が特定されない方法でのみ行われる．

3) 保有または利用する個人情報等の項目と安全管理措置および留意事項

保有または利用する個人情報は，個人の基本属性（氏名，年齢，性別，教育歴，職業歴，利き手，知的機能）や医学的情報（既往歴，現病歴），リクルートに必要な連絡先が挙げられる．また，個人の基本属性や要配慮個人情報に該当する既往歴や現病歴については，保管先を分割するなどして管理に一層の注意を払う．データの解析等は全て京都大学大学院医学研究科人間健康科学系専攻内で行うこととし，外部への流出やデータの紛失を防止するための対策をとる．

4) 研究組織全体の情報管理の責任を負う者

　　　全体の情報管理の責任は研究責任者が負うものとする．

5） 同意撤回後のデータの取り扱いについて

※①自機関内での匿名化前/後 ②解析前/後 ➂公表前/後 等時期に応じた対応

上記の①～③の公表前において，同意撤回が求められた場合，個人情報及び個人データの破棄方法は，紙媒体であればシュレッダーを行い，電子媒体であれば，データを完全に削除することで破棄を行う．公表後に同意の撤回を求められた場合には，その後の発表や公表に用いるデータから除外する．

9.　研究対象者に生じる負担並びに予測されるリスクおよび利益・総合的評価・対策

1） 負担・リスク

本研究で予想される対象者の負担及びリスクは，1日あたり最長2時間半，3日間で合計250分程度の時間的拘束が生じることや，それに伴う精神心理的負荷が挙げられる．また，Sensory Roomという閉鎖空間内にて一定時間，研究実施者と過ごし，行動を観察・記録されるという精神心理的負荷が考えられる．

ただし今回の測定に用いる機器は非侵襲的で，安全性は十分に証明されている．また，評価項目や介入内容であるSensory Roomを用いた介入や静的活動は，臨床現場において精神疾患を有する患者を対象として広く行われている侵襲性の少ない内容であり，対象者にとって耐えられる程度の比較的小さな負担であると考えられる．

2） 利益

本研究において，Sensory Roomを用いた介入や静的活動を行うことによって，研究対象者の自律神経機能や気分状態等が改善する可能性がある．

また，希望者は本研究の結果のフィードバックを得られることである．結果のフィードバックを受けることで，普段は知ることができない自らの状態を定量的に理解ができるという点で，研究対象者に利益があると考える．

3） 負担・リスクと利益の総合的評価

　負担・リスクが利益を上回るものではなくバランスが取れていると考えられる．

4） 負担・リスクを最小化する対策

研究対象者の疲労等に留意しながら適宜，休息を挿みながら実験を行うこととする．実験中は検査者側から適宜，声掛けしながら研究対象者が過度に疲労しないよう，注意深く観察しながら進行させることとする．また，計測および介入は作業療法士のみが行うこととする．万が一，体調の悪化や過度な精神心理的負荷が生じた際には，直ちに計測を中止する．このような場合には，まずバイタル測定を実施し，リハビリテーション実施基準に従って，脈拍120/分以上，拡張期血圧120mmHg以上，収縮期血圧200mmHg以上のいずれかに該当する場合は実験を中止する．バイタルに異常がみられない場合でも，負担感が強く対象者からの申し出や表情および呼吸、発汗状態等から続行による更なる体調の悪化が懸念される場合には実験を中止して研究対象から除外する．対象者の希望を伺いながら数分の休息によって負担感の回復が見込める場合には，5分程度の休息をとって計測を再開する．対象者に対して，対象者自ら研究の実施，続行を拒否する権利を有していることを周知するとともに，測定および介入中は対象者とコミュニケーションを取れる環境を常時確保する．

10.　試料・情報の保管および廃棄の方法

- 1. 情報等の保管期間

論文化後10年間保管する．

- 1. 情報等の保管方法（漏えい，混交，盗難，紛失等の防止対策）

データの解析等は全て医学研究科人間健康科学系専攻内で行うことにし，研究室内に研究用のパソコンを購入し，インターネットに接続しない状態で使用することで外部への流出やデータの紛失を防止するための対策をとる．また，パソコンにパスワードをかけることで盗難や紛失を防止する．

- 1. 保管期間後に廃棄する場合はその処理の方法

保管期間終了後および同意撤回が求められた場合，個人情報及び個人データの破棄方法は，紙媒体であればシュレッダーを行い，電子媒体であれば，データを完全に削除することで破棄を行う．

11.　試料・情報の二次利用および他研究機関への提供の可能性

本研究で収集した試料・情報は，同意を受ける時点では特定されない将来の研究のために用いる可能性がある．二次利用および他研究機関へ提供する際は，新たな研究計画について倫理審査委員会で承認された後に行う．また，対象者にはメール通知にてオプトアウトを行い，研究対象者が拒否できる機会を保障する．

12.　倫理審査委員会及び研究機関の長への報告内容および方法

・　研究の科学的合理性を損なう事実もしくは情報，または損なうおそれのある情報を得た場合は，速やかに安全性情報を提出する．

・　研究の倫理的妥当性や研究実施の適正性，研究結果の信頼性を損なう事実もしくは情報，または損なうおそれのある情報を得た場合は，速やかに不適合報告書を提出する．

・　年次報告は毎年行う．また，中止・終了報告は適宜行う．

13.　研究の資金・利益相反

1） 研究資金の種類および提供者

資金は運営費交付金(教育研究費）および科研費若手研究（22K17631）により実施する．

2） 提供者と研究者との関係

　　　　本研究は，特定の企業からの資金提供を受けていない．

3） 利益相反

利益相反について，「京都大学利益相反ポリシー」「京都大学利益相反マネジメント規程」に従い，「京都大学臨床研究利益相反審査委員会」において適切に審査している．

14.　研究対象者等およびその関係者からの相談等（遺伝カウンセリングを含む）への対応

1） 研究課題ごとの相談窓口

研究対象者全員に研究責任者の連絡先を提示し，常時連絡が取れるようにする．

本研究の研究責任者：稲富　宏之

京都大学大学院医学研究科人間健康科学系専攻脳機能リハビリテーション学分野　教授

〒606-8507　京都市左京区聖護院川原町53

2） 京都大学の相談等窓口

京都大学医学研究科 総務企画課　研究推進掛

15.　研究対象者等の経済的負担または謝礼

1） 研究参加への謝礼

評価および介入を含め，図３に示す通りに参加いただいた項目に応じた金額のQUOカードを謝礼として支払う．全ての評価および介入に参加いただいた場合，1人当たり合計で4,000円分のQUOカードを支給する．なお，予備調査にて適格基準を満たさなかった場合は，500円分のQUOカードを謝礼として支払う．


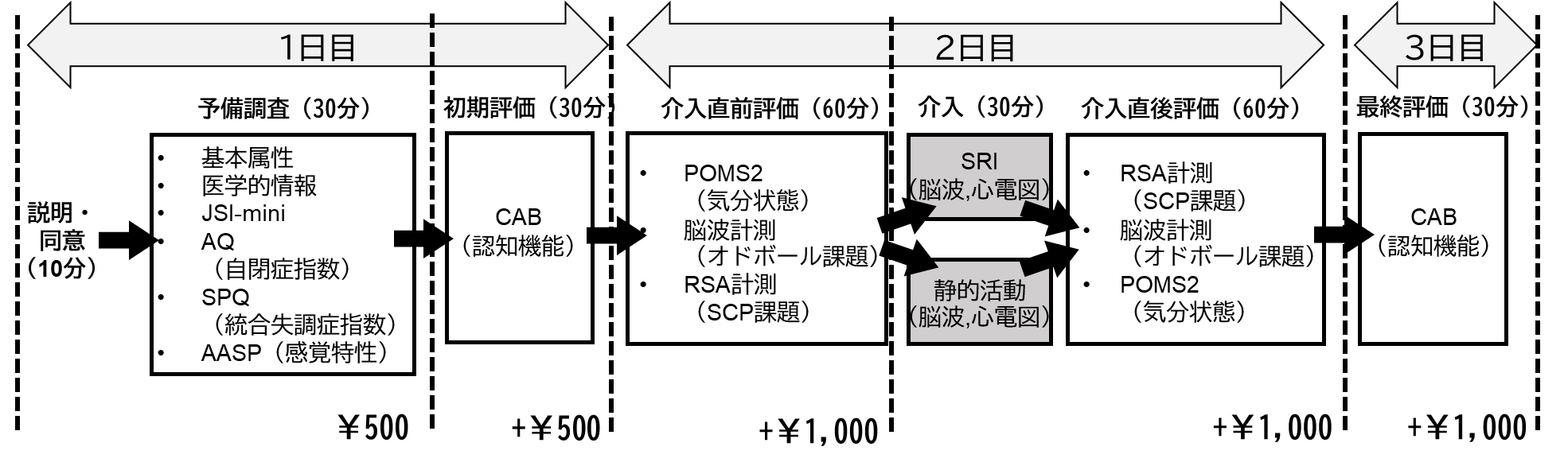


図３：謝金の内訳

16.　研究対象者に係る研究結果（偶発的所見を含む）等の取扱い

　本研究では，自閉症スペクトラム指数や統合失調症スペクトラム指数の評価によって，ASD傾向や統合失調症傾向があることが判明する可能性があるが，研究対象者への積極的なフィードバックは実施しない．理由として，本調査で用いる指数はASDや統合失調症の診断基準に直結するものではなく，あくまで点数化による行動特性の傾向を示すものであることや，ASDや統合失調症傾向があっても当人が日常生活で困難さを抱えていない場合は医療的な介入は通常必要ないとされていることが挙げられる．研究対象者から結果の開示や相談の要望があった場合は，分担研究者である精神科医をはじめ，本研究を構成する医療従事者において相談等の対応をとる．

17.　研究の実施体制（研究機関の名称および研究者等の氏名を含む）

1） 研究責任者の氏名，所属，職位，役割分担

役割：研究の企画と総括，マネジメントを行う．研究の管理と実施，個人情報の管理を行う．

稲富　宏之　京都大学大学院医学研究科人間健康科学系専攻先端リハビリテーション科学コース

先端作業療法学講座脳機能リハビリテーション学分野，教授

2） 研究実施者の氏名，所属，職位，役割分担

役割：研究の計画，実施，解析，考察，論文執筆等，研究の実施全般を行う．

大塚　日花里　京都大学大学院医学研究科人間健康科学系専攻先端リハビリテーション科学コース

先端作業療法学講座脳機能リハビリテーション学分野，博士後期課程，作業療法士

仲西　美穂　　京都大学大学院医学研究科人間健康科学系専攻先端リハビリテーション科学コース

先端作業療法学講座脳機能リハビリテーション学分野，修士課程，作業療法士

鬼塚　安純　　京都大学大学院医学研究科人間健康科学系専攻先端リハビリテーション科学コース

先端作業療法学講座脳機能リハビリテーション学分野，修士課程，作業療法士

竹渕　陸　　　京都大学医学部人間健康科学科先端リハビリテーション科学コース

先端作業療法学講座，学部3回生

木下　詩織　　京都大学医学部人間健康科学科先端リハビリテーション科学コース

先端作業療法学講座，学部3回生

出口　幸憲　　京都大学医学部人間健康科学科先端リハビリテーション科学コース

先端作業療法学講座，学部3回生

3）分担研究者の氏名，所属，職位，役割分担

役割：研究のコーディネートや計画，実施，解析，考察についての助言を行う．

古田　真里枝　　京都大学大学院医学研究科人間健康科学系専攻先端看護科学コース

　　　　　　　　先端広域看護科学講座，周産期疫学分野，教授

大滝　千文　　　京都大学大学院医学研究科人間健康科学系専攻先端看護科学コース

　　　　　　　　先端広域看護科学講座，周産期疫学分野，講師

谷向　仁　　　　京都大学大学院医学研究科人間健康科学系専攻先端リハビリテーション科学コース

先端作業療法学講座脳機能リハビリテーション学分野，准教授

入江　啓輔　　　京都大学大学院医学研究科人間健康科学系専攻先端リハビリテーション科学コース

先端作業療法学講座臨床認知神経科学分野，講師，作業療法士

森　泰祐　　　　京都大学医学部附属病院デイ・ケア診療部，作業療法士

4） 試料・情報の管理について責任を有する者の氏名，所属，職位

稲富　宏之　京都大学大学院医学研究科人間健康科学系専攻脳機能リハビリテーション学分野，教授

5） 統計解析担当者，データマネージメント担当者の氏名，所属，職位

古田　真里枝　　京都大学大学院医学研究科人間健康科学系専攻先端看護科学コース

　　　　　　　　先端広域看護科学講座，周産期疫学分野，教授

大滝　千文　　　京都大学大学院医学研究科人間健康科学系専攻先端看護科学コース

　　　　　　　　先端広域看護科学講座，周産期疫学分野，講師

18.　研究実施計画書の変更，および改訂

研究実施計画書に変更及び改訂を要する場合は，再度，倫理審査委員会に変更申請を行い，承認を得るものとする．

19.　遵守すべき倫理指針

本研究は「ヘルシンキ宣言」と「人を対象とする生命科学・医学系研究に関する倫理指針」に基づき実施する．

20.　研究成果の帰属

本研究の成果は，京都大学に属するものとする．

21.　参考文献

1. Thye, M. D., Bednarz, H. M., Herringshaw, A. J., Sartin, E. B., & Kana, R. K. (2018). The impact of atypical sensory processing on social impairments in autism spectrum disorder. Developmental cognitive neuroscience, 29, 151-167.
2. Green, M. F., Horan, W. P., & Lee, J. (2015). Social cognition in schizophrenia. Nature Reviews Neuroscience, 16(10), 620-631.
3. Sutton, D., Wilson, M., Van Kessel, K., & Vanderpyl, J. (2013). Optimizing arousal to manage aggression: A pilot study of sensory modulation. International journal of mental health nursing, 22(6), 500-511.
4. Burns, P., & Van Der Meer, R. (2021). Happy Hookers: findings from an international study exploring the effects of crochet on wellbeing. Perspectives in public health, 141(3), 149-157.
5. Shimada, T., Ohori, M., Inagaki, Y., Shimooka, Y., Sugimura, N., Ishihara, I., ... & Kobayashi, M. (2018). A multicenter, randomized controlled trial of individualized occupational therapy for patients with schizophrenia in Japan. PLoS One, 13(4), e0193869.
6. DeBoth, K. K., Reynolds, S., Lane, S. J., Carretta, H., Lane, A. E., & Schaaf, R. C. (2021). Neurophysiological Correlates of Sensory-Based Phenotypes in ASD. Child Psychiatry & Human Development, 1-13.
7. Rissling, A. J., & Light, G. A. (2010). Neurophysiological measures of sensory registration, stimulus discrimination, and selection in schizophrenia patients. Behavioral neurobiology of schizophrenia and its treatment, 283-309.
8. Yasuma, F., & Hayano, J. I. (2004). Respiratory sinus arrhythmia: why does the heartbeat synchronize with respiratory rhythm?. Chest, 125(2), 683-690.
9. Zlotnik, S., Attias, J., Pratt, H., & Engel-Yeger, B. (2018). Neurophysiological manifestations of auditory hypersensitivity correlate with daily life experiences. Neuroscience and Medicine, 9(01), 29.
10. van den Boogert, F., Spaan, P., Sizoo, B., Bouman, Y. H., Hoogendijk, W. J., & Roza, S. J. (2022). Sensory Processing, Perceived Stress and Burnout Symptoms in a Working Population during the COVID-19 Crisis. International Journal of Environmental Research and Public Health, 19(4), 2043.
11. Sulik, M. J., Eisenberg, N., Spinrad, T. L., & Silva, K. M. (2015). Associations between respiratory sinus arrhythmia (RSA) reactivity and effortful control in preschool‐age children. Developmental psychobiology, 57(5), 596-606.
12. Tininenko, J. R., Measelle, J. R., Ablow, J. C., & High, R. (2012). Respiratory control when measuring respiratory sinus arrhythmia during a talking task. Biological psychology, 89(3), 562-569.
13. Schaaf, R. C., Benevides, T. W., Blanche, E., Brett-Green, B. A., Burke, J., Cohn, E., ... & Schoen, S. A. (2010). Parasympathetic functions in children with sensory processing disorder. Frontiers in integrative neuroscience, 4, 4.
14. Chalmers, A., Harrison, S., Mollison, K., Molloy, N., & Gray, K. (2012). Establishing sensory-based approaches in mental health inpatient care: a multidisciplinary approach. Australasian Psychiatry, 20(1), 35-39.
15. Wiglesworth, S., & Farnworth, L. (2016). An exploration of the use of a sensory room in a forensic mental health setting: Staff and patient perspectives. *Occupational Therapy International*, *23*(3), 255-264.
16. Koshiyama, D., Kirihara, K., Tada, M., Nagai, T., Fujioka, M., Usui, K., ... & Kasai, K. (2020). Reduced auditory mismatch negativity reflects impaired deviance detection in schizophrenia. Schizophrenia bulletin, 46(4), 937-946.
