# Supplementary material for: Effects of sensory room intervention on autonomic function in healthy adults: A pilot randomized controlled trial
Source: PLoS One. 2025 Apr 23;20(4):e0319649. doi: 10.1371/journal.pone.0319649 (PMC12017487; doi:10.1371/journal.pone.0319649)
Supplement: S3 File — (DOCX) [file pone.0319649.s003.docx]

research plan

November 29, 2022 Ver.1

December 6, 2022 Ver.2

December 23, 2022 Ver.3

February 2, 2023 Ver.4

February 21, 2023 Ver.5

February 28, 2023 Ver.6

1. Title of Research

　　Verification of the effects of an intervention using the Sensory Room on sensory processing characteristics in healthy adults: a randomized controlled trial

2. Background of the study

In recent years, difficulties in social participation have become an issue for people with psychiatric disorders such as schizophrenia and autism spectrum disorder (ASD), with sensory processing disorder considered one of the causes^1)^. Sensory processing disorder refers to the process of perceiving all stimuli in the environment as visual, auditory, and other information, integrating the information in the brain, recognizing the situation, and connecting it to the next action. This is believed to result in hypersensitive reactions to environmental stimuli and misunderstanding of social signs such as facial expressions shown by others, as well as social withdrawal resulting from such reactions^2)^.

In clinical settings, interventions using sensory rooms that focus on sensory processing disorders are commonly used in Western countries. sensory rooms are rooms in which sensory stimuli can be modulated to the appropriate level, and their effects are said to include relaxation of the body and mind and the acquisition of self-control over sensory input^3)^. However, most of the reports are based on qualitative studies, and the evidence is insufficient to verify them.

Conventional psychiatric occupational therapy involves programs using a variety of occupational activities and exercise, such as handicrafts, reading, puzzles, and light exercise. Interventions using occupational activities are said to have a certain effect on improving mood and cognitive function^4)5)^. However, none of these interventions focus on the senses, and the difference in effectiveness between occupational activities and sensory interventions is not clear.

In the field of neurophysiology, it has been shown that sensory processing disorders are associated with autonomic function and electroencephalography. Specifically, children with ASD have reduced parasympathetic function when exposed to sensory stimuli, resulting in a disturbance in the balance of autonomic function^6)^, and patients with schizophrenia have impaired cognitive processes such as paying attention to information detected after inputting sensory stimuli, as indicated by the event-related potentials (ERPs) of the electroencephalogram, N1, MMN (mismatch negativity), and P300^7)^. For parasympathetic function, we focused on respiratory sinus arrhythmia (RSA), which is a heart rate variability that is faster during inspiration and slower during expiration, reflecting vagal activity that fluctuates with respiration and is widely used as an indicator of pure parasympathetic function. It is widely used as an indicator of pure parasympathetic function^8)^.

Atypical sensory processing characteristics are also present in healthy individuals who have not been diagnosed with psychiatric disorders. In a study of healthy subjects, it was reported that latencies of P2 event-related potentials to large frequency changes in auditory stimuli were significantly longer in hypersensitive subjects than in nonsensitive subjects^9)^ and that hypersensitivity to sensory stimuli and the tendency to avoid sensory stimuli were associated with the amount of stress felt in daily life^10)^.

However, as far as we could find, there were no reports on the usefulness of the Sensory Room using physiological indices such as autonomic function or electroencephalogram (EEG) activity, regardless of the diagnosis of psychiatric disorders, and the effect of the Sensory Room at the biological response level has not been clarified.

3. Purpose and Significance of the Study

1. What are you trying to clarify?

The purpose of this study is to demonstrate the effectiveness of a sensory room intervention (hereafter referred to as SRI) focused on sensory processing characteristics in healthy subjects through changes in biological indicators such as autonomic indices and electroencephalogram, as well as subjective mood state and cognitive function. This will allow us to clarify the usefulness of the intervention focusing on sensory processing characteristics through basic research.

The research question is: Does a sensory room intervention increase parasympathetic activity and EEG amplitude, and improve mood state and cognitive function compared to static activity in healthy adults?

1. What is the medical and social significance?

The uniqueness of this study lies in demonstrating the therapeutic effects of the Sensory Room through changes in physiological indices, such as autonomic nervous system function and EEG activity, as well as cognitive function. We believe that demonstrating the usefulness of the Sensory Room in healthy subjects through objective measures and improved cognitive function will greatly contribute to proposing a new treatment method for people with psychiatric disorders, of which a high percentage are believed to suffer from sensory processing disorders. In the future, the effectiveness of the Sensory Room is expected to be widely publicized, leading to its installation in hospitals, educational institutions, and workplaces, and to the improvement of mental health care and work efficiency for people with developmental disabilities and mental disorders as well as healthy people. In addition, the role of occupational therapists and the usefulness of occupational therapy in making the above contributions will be clarified.

4. How to select research subjects

1. setting

We will collect the subjects by posting information and distributing flyers (Ref.1) to healthy adults, undergraduate and graduate students living in the community, and others who inquire about this research, explaining the contents of this research orally and in writing.

1. Eligibility Criteria

　　Persons who meet all of the following selection criteria and none of the exclusion criteria will be enrolled as eligible.

Selection Criteria

1. The patient must be between 18 and 65 years of age at the time of registration.
2. Written consent to participate in the research has been obtained from the research subjects themselves.
3. Total score of 1 or more points on the JSI-mini (Japanese Sensory Inventory mini) (Ref.2)

The rationale for setting the age of the subjects (a) is that the subjects of this study are adults and that the decline in physical and mental functions due to old age may affect the results of the study. The reason for (c) was to target people who have some characteristics of sensory processing in their daily lives, which is the intervention target of this study.

Exclusion Criteria

Persons who fall into any of the following categories shall not be used as research subjects.

1. Complications of cardiac disease.
2. Cardiac pacemaker users
3. History of epilepsy
4. Visual acuity (correctable) less than 0.7 in both eyes
5. Air conduction hearing greater than 30㏈.
6. There is tactile hypoesthesia (unable to perceive Semmes-Weinstein monofilament no. 2.83)
7. Perceived significant taste and olfactory abnormalities
8. History of any other visual or hearing impairment, language comprehension disorder, mental disorder, or motor dysfunction that would interfere with the performance of the research task.

The reasons for setting (a) and (b) are because they may affect autonomic measurements and (c) because they may affect electroencephalography measurements. The reason for (d) through (g) is to exclude people with functional abnormalities in the sensory organs to be evaluated and intervened in this study.

Suspension Criteria

1. When the research subject wishes to discontinue
2. When it is deemed appropriate to discontinue other activities.

3) Number of research subjects and rationale for setting the number of research subjects

The target number of patients for this study is 58, consisting of 29 in the intervention group and 29 in the control group.

Regarding the rationale for the setting, this study will conduct a two-group comparison (unpaired t-test, one-tailed) of the intervention and control groups for changes in the parasympathetic index (RSA: respiratory sinus arrhythmia) before and after the intervention as the main outcome. Using G*Power 3.1.9.2 and referring to previous studies^11)12)^ that utilized RSA, the effect size was set to 0.7, with an alpha error of 0.05 and a beta error of 0.20, resulting in a total sample size of 52 participants. In addition, considering the possibility of approximately 10% dropout, approximately 58 subjects will be needed for the study. 58 subjects will be randomly divided into 29 in the SRI group and 29 in the control group.

The number of subjects in the previous study^6)13)^, which investigated parasympathetic indices and sensory processing characteristics in children with ASD and normal children, was 50 and 83, respectively, and we believe that the above number of subjects is generally appropriate.

5. Method of the study and the scientific rationale for the study

1) Design

□ Intervention study

Phase of study: Exploratory study

Method of comparison: Before/after comparison/parallel group comparison

Method of allocation of intervention: Randomization

Blinding of evaluators: Blinding of only the researcher in charge of statistical analysis.

Therefore, this study is a randomized controlled trial.

2) Method

Those who are willing to participate in the study, agree to participate after receiving a thorough oral and written explanation from the investigator, and meet the eligibility criteria will be enrolled as study subjects. Using an allocation chart, the study administrator will randomly assign the subjects to the SRI group or the control group and implement the intervention. Afterwards, only the subject's ID and data will be sent to the co-researcher in charge of statistical analysis, who will conduct the analysis. In this way, blinding the researcher in charge of statistical analysis minimizes bias and ensures appropriate results.

The flow of implementation is shown in Figure 1. The study consists of six phases: (a) preliminary investigation (screening), (b) initial evaluation, (c) evaluation immediately before the intervention, (d) intervention, (e) evaluation immediately after the intervention, and (f) final evaluation. Each phase is described below. All surveys, evaluations, and interventions will be conducted at Human Health Science, Graduate School of Medicine, Kyoto University.


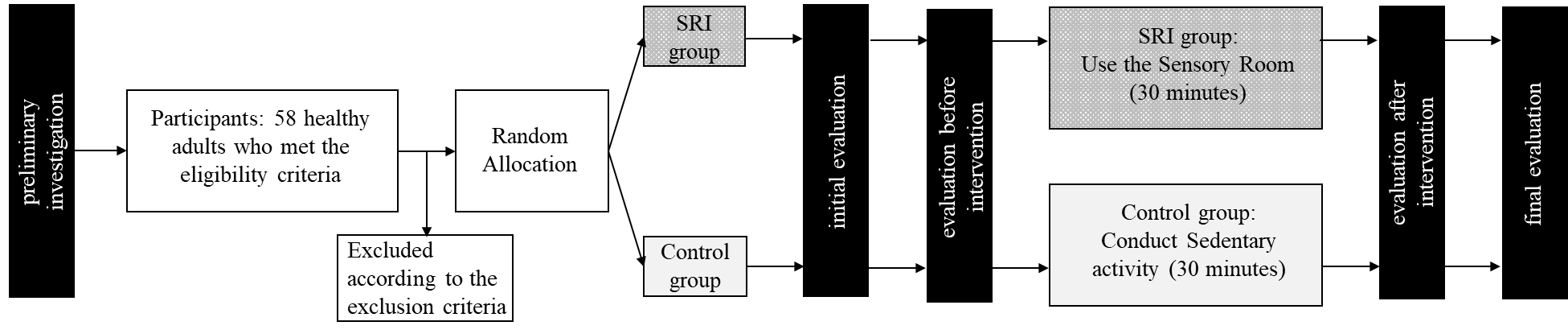


Figure 1: Flow of Implementation

1. Preliminary investigation (screening)

The main purposes of this survey are to screen subjects who are not suitable for the study and to evaluate their psychiatric disorders and sensory processing characteristics. The items measured were (1) basic information of the subjects, (2) medical information, (3) JSI-mini (Japanese Sensory Inventory mini), (4) Autism spectrum Quotient (AQ), (5) Schizotypal Personality Questionnaire (SPQ), and (6) the Japanese Adolescent/Adult Sensory Profile (A/ASP) (see "3) Observation Items and Schedule"). If the eligibility criteria are not met, the subjects will be asked to refrain from further participation in the study, and the experiment will be terminated with an honorarium, based on the time spent in the preliminary investigation. After enrollment of study subjects who meet the eligibility criteria, the subjects will be randomly assigned to two groups, the SRI group and the control group, and will be blinded to the assigned researcher (in charge of statistical analysis) until the end of the analysis.

1. Initial assessment

Following the preliminary survey, participants will be asked to participate in an initial evaluation. In the initial evaluation, you will be asked to complete (1) Cognitive Assessment Battery (CAB) . (See "3) Observation Items and Schedule"). This will serve as a standard for the temporal assessment of cognitive function of the study subjects before the intervention. This assessment will be conducted on the day before the intervention in order to reduce the burden on the research subjects and to provide potential control data for future multi-week intervention studies on people with mental disorders.

1. Evaluation before the intervention

　On the day following the initial evaluation, participants will participate in a pre-intervention evaluation before the intervention. The evaluation items will be (1) RSA measurement during the Sensory Challenge Protocol (SCP), (2) EEG measurement, and (3) Profile of Mood States 2nd Edition (POMS2). Because the RSA measurement, which is the main outcome of this study, will be conducted at the time point closest to the intervention, the measurements will be conducted in the order of POMS2, EEG, and RSA measurements. These assessments will be used as the basis for a longitudinal evaluation of the subjects' autonomic nervous system function and EEG waveform trends and subjective mood state before the intervention.

1. Intervention

The SRI (Sensory Room Intervention) group will receive an intervention using a sensory room, and the control group will receive 30 minutes of static activity. Of the 58 subjects in the study, approximately 20 subjects who gave their consent will undergo EEG, electrocardiogram (ECG), and pulse wave measurements to determine the degree of relaxation, cognitive activation, and sleep arousal during the intervention (Ref. 13).

≪ Intervention details.≫

1. SRI: A Sensory Room of approximately 5.0 m^2^ x 2.0 m will be created in the Department of Human Health Science building of the University, and the subject and the researcher will enter the room one by one. The researcher who enters the room must be a licensed occupational therapist. Inside the room are dimmable and color-controlled lights, a bubble tube, a music player with healing music, a weighted blanket, beaded cushions, an aroma diffuser with aroma oil, and a tactile ball. At the beginning, the researcher suggests sensory stimuli based on the subject's A/ASP score according to his/her sensory processing characteristics, and gradually encourages the subject to select and adjust sensory stimuli on his/her own. The intervention time is 30 minutes, based on a previous study that used a sensory room intervention^14)15)^. At the end of the intervention, the subjects were asked about their impressions of the items in the Sensory Room using a simple questionnaire and the NASA-TLX (Task Load Index) (Ref. 11).
2. Sedentary activity: The participants will be asked to perform creative activities such as knitting and origami, and low-intensity activities of less than approximately 3 METs (Ref. 12) such as puzzles, reading, and watching movies in a chair-sitting position. The location will be the same sensory room as in ①, but the subject will be in a lighted room without using the items used in ① or other items that stimulate the subject's awareness of sensory stimuli. The subject and the researcher will enter the room one by one, and the subject will be asked to choose freely from the work activities in the room. 30 minutes will be allotted for each intervention, as in SRI. At the end of the intervention, the subjects are asked about their impressions of the activities using a simple questionnaire or the NASA-TLX (Ref. 11).
3. Evaluation after the intervention

After the 30-minute intervention, a post-intervention evaluation was conducted. The evaluation items consist of (1) RSA measurement during the Sensory Challenge Protocol (SCP), (2) EEG measurement, and (3) Profile of Mood States 2nd Edition (POMS2), the same as the evaluation immediately before the intervention (see "3) Observation Items and Schedule"). The order of the procedures is RSA measurement, EEG measurement, and POMS2, in that order. The time required is approximately 60 minutes. We will examine how the intervention changes the subjects' autonomic nervous system function, EEG waveform trends, and subjective mood state in the intervention and control groups, respectively.

1. Final Evaluation

The day after the immediate post-intervention evaluation, the final evaluation will be conducted. The final evaluation will be conducted using the CAB (1) conducted in the initial evaluation (see "3) Observation Items and Schedule"). The time required is approximately 30 minutes. We will examine how the intervention changed the cognitive function of the subjects in the intervention group and the control group.

3) Observation, inspection, investigation, and reporting items and schedule

- Measurement item, measurement method, person or organization

The measurement items and methods are described below. All measurements are performed by the researcher.

1. Items to be conducted in the preliminary investigation

The following will be heard and evaluated. The time required is 30 minutes.

1. Basic information of the subjects (age, gender, educational and occupational history, handedness, intellectual function) (Ref. 3): The Japanese version of the FLANDERS Handedness Test (Ref. 4) is used to assess handedness, and the JART (Japanese Adult Reading Test) (Ref. 5) to assess intellectual function.
2. Medical information (history, current medical history, visual acuity, hearing, touch, taste, and smell) (Ref. 3): Visual acuity was assessed using the International Standardized Visual Acuity Test at 5 m, hearing using the Mimi Hearing Test, and touch using the Semmes-Weinstein monofilament.
3. JSI-mini (Japanese Sensory Inventory mini) (Ref. 2): A simplified version of the JSI-R (Japanese Sensory Inventory Revised), with 20 self-administered items. It is a simple way to grasp the tendency to receive sensory stimuli.
4. Autism spectrum Quotient (AQ) Japanese version (Ref. 6): A self-response questionnaire to assess individual autistic tendencies in adults with normal intelligence. It consists of 10 questions in each of the five domains (social skills, attention switching, attention to detail, communication, and imagination) that indicate symptoms that characterize autistic disorder, for a total of 50 items. Respondents were asked to select one of the following four options: "yes," "somewhat agree," "somewhat disagree," or "no." The total score ranged from 0 to 50 points. The total score ranges from 0 to 50, with a cutoff of 33 or higher.
5. Schizotypal Personality Questionnaire (SPQ) Japanese version (Ref. 7): A self-administered questionnaire that comprehensively measures schizotypal personality characteristics in healthy subjects. There are 74 items in total, with "yes" and "no" answers.
6. Japanese Adolescent/Adult Sensory Profile (A/ASP) (Ref. 8): A self-administered questionnaire that rates sensory processing tendencies in terms of "low registration," "sensory seeking," "sensory sensitivity," and "sensory avoidance. The subjects were between 11 and 82 years old. There are 60 items with 15 items each, and the range of possible scores for each of the four characteristics is between 15 and 75 points.
7. Items to be performed in the initial and final evaluation

The following will be evaluated. The time required is about 30 minutes.

1. Cognitive Assessment Battery (CAB) (Ref. 9): Measures function in various cognitive domains such as attention, concentration, perception, memory, executive function, and coordination, as well as physical, psychological, and social well-being. The assessment is conducted online.
2. Items to be performed in the evaluation immediately before and immediately after the intervention

The following will be evaluated. The time required is about 60 minutes.

1. RSA measurement during the Sensory Challenge Protocol (SCP): The SCP consists of four phases: (1) a resting period (3 min), (2) a task period during which each of six sensory stimuli (visual, auditory, etc.) is presented, (3) a recovery period (3 min), and (4) sustained auditory stimulation (2 min). The RSA was measured using the PolyPul (Nihonsanteku) portable pulse wave measurement device.
2. EEG measurement: EEG is measured by presenting a task consisting of an oddball task and a Many-standards task^16)^ . The oddball task consists of high-frequency standard stimuli and low-frequency deviant stimuli. The auditory stimuli are designed in the order of frequency and probability of presentation: 1000 Hz, 80% for the standard stimuli, and 1100 Hz, 20% for the deviant stimuli. The Many-standards task has more combinations of frequencies and durations of auditory stimuli than the Oddball task, and the appearance of stimuli is more difficult to predict. The electrode positions are set according to the international 10-20 method, and the sampling frequency is set to 1000 Hz.
3. Profile of Mood States 2nd Edition (POMS2) Japanese version Online version (Ref. 10): A questionnaire designed to assess mood states such as anger, confusion, and depression. It consists of seven scales: Anger-Hostility, Confusion-Bewilderment, Depression-Dejection, Fatigue-Inertia, Tension-Anxiety, Vigor-Activity, and Friendliness. The total mood disturbance (TMD) score, which is a composite of these seven scales, is also calculated. Two types of mood states can be assessed: persistent mood (how the person felt in the past week) and transient mood (how the person feels at the present moment). An all-item version (65 items) for adults will be used. The survey takes approximately 8 to 10 minutes.
4. Items to be retrieved during intervention

　The following items will be obtained during the intervention in approximately 20 subjects who gave consent. The sampling frequency is 1000 Hz.

1. EEG measurement: EEG during the intervention will be measured from 32 electrodes, and the power spectrum of each band will be calculated for each site by frequency analysis using Fast Fourier Transform. The measurement equipment used will be an actiCHamp Plus (PHSIO-TECH), and electrode positions followed the international 10-20 method.
2. ECG and pulse wave measurement: ECG electrodes are guided at three points (right subclavian, left subclavian, and lower chest), and RSA values are calculated from the obtained waveforms. The measurement devices used are actiCHamp Plus (PHSIO-TECH) or PolyPul (Nihonsanteku), a portable pulse wave measuring device.

- Measurement Schedule:

The experimental flow is shown in Figure 2. The evaluation and intervention will be conducted over three days: on the first day, after explanation and consent, a preliminary survey and initial evaluation will be conducted; on the second day, the evaluation immediately before the intervention, the intervention, and the evaluation immediately after the intervention will be conducted; and on the third day, the final evaluation will be conducted. Each evaluation item will be conducted with appropriate breaks in between.


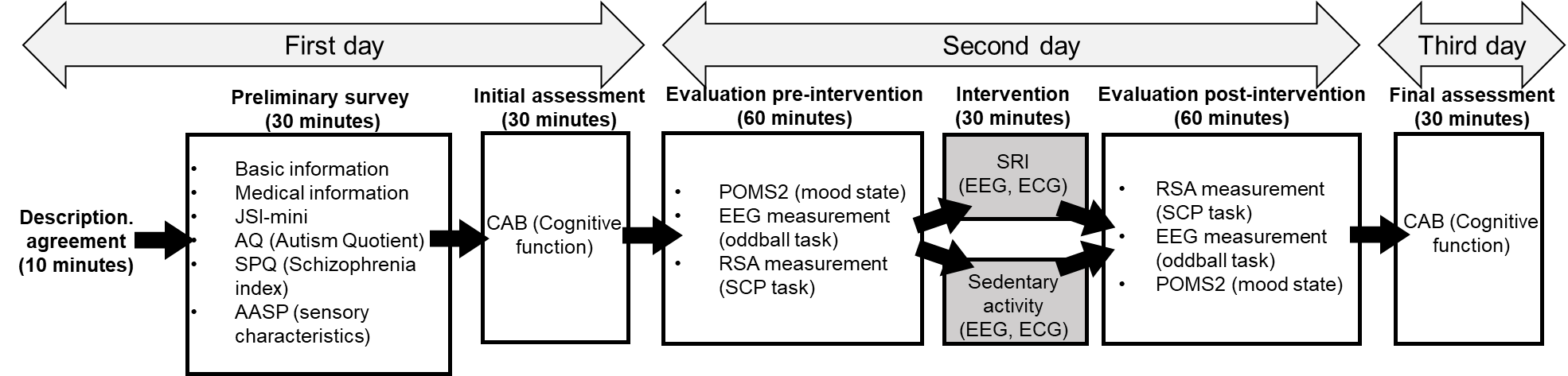


Figure 2: Measurement schedule

4) Summary of analysis

- - Primary Endpoint

(a) Differences in the variability of RSA values due to sensory stimulation of SCP before and after the intervention.

- - Secondary endpoint

(a) EEG amplitude differences before and after intervention, (b) POMS2 score differences before and after intervention, (c) CAB score differences at initial and final assessment, (d) correlation between AQ scores and RSA values, (e) correlation between SPQ scores and RSA values, (f) correlation between A/ASP scores and RSA values, (g) power spectrum by EEG band during intervention, (h) RSA power spectrum of each EEG band, (h) RSA during intervention

- - Main Analysis Methods

The statistical significance level is set at p < 0.05. A one-tailed test is used.

Basic information, medical information, and observation records during the use of the sensory room were compiled as descriptive statistics: RSA values were calculated as the difference between the SCP resting period and the mean value during each sensory stimulus; EEG amplitudes were obtained by subtracting the standard stimulus waveform from the deviant stimulus waveform of the presented task to obtain the amplitude of the positive or negative component during latencies of approximately 200 to 400 ms. The EEG amplitude is obtained by subtracting the standard stimulus waveform from the deviant stimulus waveform of the presented task.

Unpaired t-tests will be conducted between the two groups to confirm the absence of any differences between the SRI and control groups in the baseline pretest, initial assessment, and the assessment immediately before the intervention. Next, we will compare the differences in RSA values, EEG amplitude, and POMS2 scores between the two groups at the immediately pre-intervention and immediately post-intervention assessments using an unpaired t-test. The differences in CAB scores between the initial and final assessments will be compared between groups using an unpaired t-test. If there is a significant difference between the two groups in the items measured at baseline, stratified or subgroup analyses will be conducted.

Additionally, correlation coefficients will be determined for the association between the AQ, SPQ, and A/ASP scores from the preliminary study and the differences in RSA values, EEG amplitude, and POMS2 scores at the time of the evaluation immediately before and immediately after the intervention. For cerebral blood flow, the difference between the mean values at the beginning and end of each 5-minute period in the two groups at the beginning and end of the intervention will be calculated and compared between the groups using an uncorrelated t-test.

For the measures during the intervention, t-tests with no correspondence between groups will be performed for the power spectrum per band of the EEG and the RSA.

6. Research period

1) Research subject enrollment period

　Four years from the date of approval by the head of the research institution.

2) Study subjects observation period

　　　The observation period for research subjects will be 3 days from the time of the preliminary survey.

3) Research implementation period

The research period is five years from the date of approval by the head of the research institution.

7. Procedure for obtaining informed consent ("IC")

Before the preliminary study, the subject will be fully informed about the study using a written explanation of the study. The measurement items, measurement time, intervention details, and non-invasive nature of the study will be explained, and the subject will then decide to participate of his/her own free will. The subject's consent to participate in the study and his/her signature constitute the informed consent procedure.

8. Handling of personal information, etc.

1) Types of personal information, such as materials and information handled in the research

　　This study deals with pseudonymized information.

2) When and how to create 1)

The data and other personal information are collected by the principal investigator, and the subject's name is converted to an ID number to create a pseudonym. The principal investigator in charge of the analysis will conduct the analysis using the data whose names have been converted into ID numbers. The correspondence list containing the names and IDs will be kept in a locked vault or on a non-transferable PC with no network connection to the outside world for 10 years after the publication of the paper, to ensure that the data is not leaked to the outside world and that the privacy of the subjects is fully considered. Research results may be published in academic journals or presented at academic conferences, but only in a manner that strictly protects the privacy of individuals and does not identify them.

3) Items of personal information, etc. to be retained or used, security control measures and points to note

Personal information to be retained or used includes basic personal information (name, age, gender, education, occupation, handedness, intellectual function), medical information (medical history, current medical history), and contact information necessary for recruitment. In addition, we will pay more attention to the management of the basic personal information and the medical history and current medical history that fall under the category of sensitive personal information, for example, by dividing the storage location of such information. All data analysis will be conducted within the Department of Human Health Sciences, Graduate School of Medicine, Kyoto University, and measures will be taken to prevent data loss and leakage to outside parties.

4) A person responsible for information management of the entire research organization

　　　Responsibility for overall information management rests with the principal investigator.

5) Handling of data after withdrawal of consent

※(1) Before/after anonymization within own institution (2) Before/after analysis (3) Before/after publication, etc., depending on the timing.

If a withdrawal of consent is requested before the publication of the above items (1) through (3), the personal information and data will be destroyed by shredding if it is on paper media, or by completely deleting the data if it is on electronic media. If withdrawal of consent is requested after publication, the data will be excluded from subsequent presentations and publications.

9. Burdens and anticipated risks and benefits to the research subjects, and the overall assessment and measures to be taken

1) Burdens and risks

The burden and risk to the subjects in this study include the time commitment of up to 2.5 hours per day, or about 250 minutes in total over three days, and the accompanying psychosomatic burden. In addition, the subjects will spend a certain amount of time in a closed space called a "sensory room" with the researcher, and their behavior will be observed and recorded, which is considered to be a psychosomatic burden.

However, the instruments used in this study are noninvasive, and their safety is well documented. Additionally, the evaluation items and interventions, such as the intervention using the Sensory Room and sedentary activity, are not invasive and are widely used in clinical settings for patients with psychiatric disorders, and are considered to be relatively small burdens that the subjects can tolerate.

2) Profit

In this study, the intervention using the Sensory Room and sedentary activity may improve the autonomic nervous system function and mood state of the study subjects.

In addition, those who wish to do so can obtain feedback on the results of this study. We believe that receiving feedback on the results will benefit the research subjects in that they will be able to quantitatively understand their own condition, which is not normally available to them.

3) Comprehensive evaluation of burdens, risks and benefits

　The burdens and risks do not outweigh the benefits and are considered to be in balance.

4) Measures to minimize burdens and risks

The experiment should be conducted while inserting rest breaks as appropriate, paying attention to research subject’s level of fatigue. During the experiment, the examiner should call out to the subject as necessary, while carefully observing the subject so that the subject does not become excessively fatigued. Only occupational therapists are to perform measurements and interventions. In the unlikely event of deterioration of physical condition or excessive psychosomatic stress, the measurement will be stopped immediately. In such cases, vital measurements should be taken first, and the experiment should be stopped if the pulse rate is 120/min or higher, diastolic blood pressure is 120 mmHg or higher, or systolic blood pressure is 200 mmHg or higher, in accordance with the rehabilitation implementation criteria. Even if there is no abnormality in the vital signs, the experiment will be terminated and the subject will be excluded from the study if there is concern that their physical condition will deteriorate further due to the continuation of the experiment, based on the subject's strong sense of burden, facial expression, respiration, sweating, and so on. If the subject's sense of strain is expected to recover after a few minutes of rest, we will resume the measurement after about 5 minutes of rest, while listening to the subject's wishes. The subject should be informed that he/she has the right to refuse to continue the study, and an environment in which he/she can communicate with the subject should be maintained at all times during the measurement and intervention.

10. Method of storage and disposal of samples and information

- 1. Retention period of information, etc.

Retain the paper for 10 years after publication.

- 1. Methods of storing information, etc. (measures to prevent leakage, mixing, theft, loss, etc.)

All data analysis, etc., will be performed within the Department of Human Health Sciences, Graduate School of Medicine. A computer for research use will be purchased in the laboratory and used without Internet access to prevent leakage to the outside and loss of data. In addition, passwords will be applied to the computers to prevent theft or loss of data.

- 1. If disposed of after the storage period, the method of disposal

After the retention period ends or when consent is withdrawn, personal information and personal data are destroyed by shredding paper media or by completely deleting electronic media.

11. Possibility of secondary use of samples/information and provision to other research institutions

Samples and information collected in this study may be used for future research that is not identified at the time consent is obtained. Secondary use or provision to other research institutions will be conducted after approval of a new research plan by the Ethics Review Committee. In addition, subjects will be notified by e-mail to opt out, and they will be guaranteed the opportunity to refuse.

12. Contents and methods of reporting to the Ethical Review Committee and the head of the research institution

We promptly submit safety information if we obtain facts or information that undermine or may undermine the scientific rationale for the research.

If we obtain facts or information that undermine or may undermine the ethical validity of the research, the appropriateness of the conduct of the research, or the reliability of the research results, we will promptly submit a Nonconformity Report.

Annual reports shall be made annually. Reports on discontinuation and termination should be made as appropriate.

13. Funding and conflicts of interest in research

1) Type of research funding and providers

Funds are provided by Grant-in-Aid for Education and Research and Grant-in-Aid for Young Scientists (22K17631) .

2) Relationship between the donor and the researcher

　　　　This study was not funded by any specific company.

3) Conflicts of Interest

Conflicts of interest are appropriately reviewed by the Kyoto University Clinical Research Conflict of Interest Review Committee in accordance with the Kyoto University Conflict of Interest Policy and the Kyoto University Conflict of Interest Management Regulations.

14. Consultation and other services (including genetic counseling) from research subjects and other related parties

1) Consultation service for each research project

Provide all research subjects with contact information for the principal investigator so that he or she can be reached at all times.

Principal investigator of this study: Hiroyuki Inadomi

Professor, Department of Brain Function Rehabilitation, Human Health Science, Graduate School of Medicine, Kyoto University

53 Shogoin Kawahara-cho, Sakyo-ku, Kyoto 606-8507, Japan

2) Kyoto University Consultation Service

Research Promotion Office, General Affairs and Planning Division, Graduate School of Medicine, Kyoto University

15. Financial burden or honorarium for research subjects, etc.

1) Gratuities for research participation

QUO cards will be paid as rewards according to the number of participants as shown in Figure 3, including both evaluations and interventions. Each participant will receive a QUO card worth a total of 4,000 yen if they participate in all evaluations and interventions. If the eligibility criteria are not met in the preliminary survey, a QUO card worth 500 yen will be paid as an honorarium.


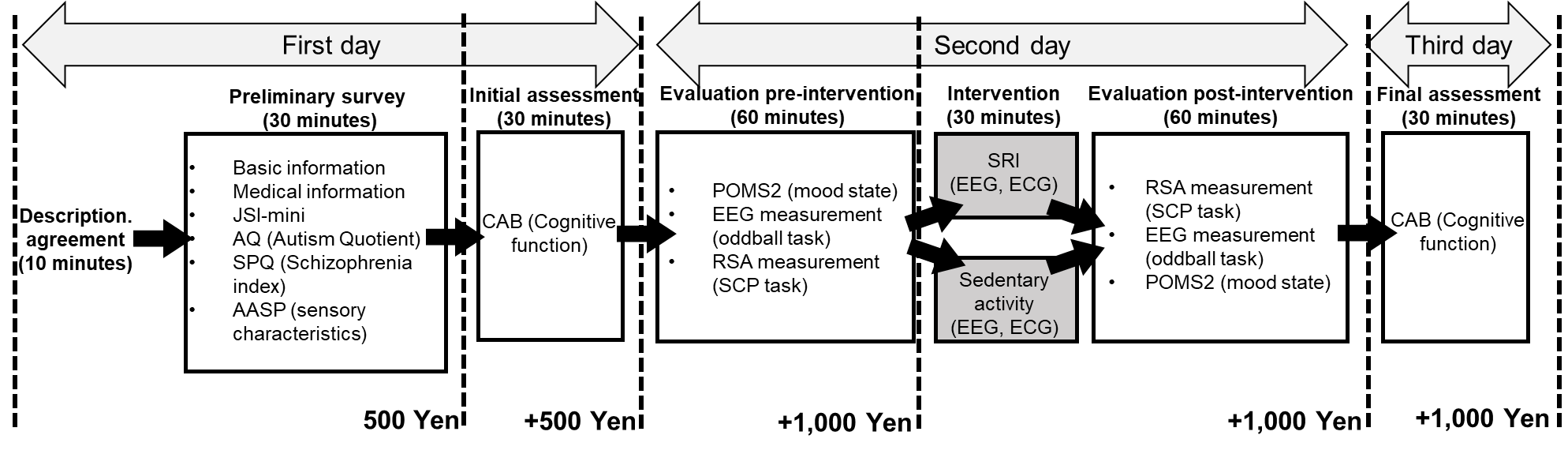


Figure 3: Breakdown of gratuities

16. Handling of research results (including incidental findings), etc., pertaining to research subjects

　In this study, the evaluation of the autism spectrum index and schizophrenia spectrum index may reveal the presence of ASD tendency or schizophrenia tendency, but active feedback to the study subjects will not be conducted. This is because indices used in this study are not directly related to the diagnostic criteria for ASD or schizophrenia, but only indicate trends in behavioral characteristics by scoring, and that medical intervention is usually not necessary when individuals with ASD or schizophrenia tendencies do not have difficulties in their daily lives. If the subjects of the study request disclosure of the results or consultation, the psychiatrist, who is a co-researcher, and other medical professionals who are part of this study will provide consultation and other responses.

17. Research implementation structure (including the name of the research institution and names of researchers, etc.)

1) Name, affiliation, position and role of the principal investigator

Role: To plan, oversee and manage research. Manage and conduct the research, and manage personal information.

Hiroyuki Inadomi

Professor, Human Health Sciences, Graduate School of Medicine, Course of Advanced Rehabilitation Science, Department of Advanced Occupational Therapy, Department of Brain Function Rehabilitation, Kyoto University

2) Name, affiliation, position, and role of the person conducting the research

Role: To plan, conduct, analyze, discuss, and write papers on the research.

Hikari Otsuka

Ph.D. student, Occupational Therapist, Human Health Sciences, Graduate School of Medicine, Course of Advanced Rehabilitation Science, Department of Advanced Occupational Therapy, Department of Brain Function Rehabilitation, Kyoto University

Miho Nakanishi

Master's student, Occupational Therapist, Human Health Sciences, Graduate School of Medicine, Course of Advanced Rehabilitation Science, Department of Advanced Occupational Therapy, Department of Brain Function Rehabilitation, Kyoto University

Azumi Onitsuka

Master's student, Occupational Therapist, Human Health Sciences, Graduate School of Medicine, Course of Advanced Rehabilitation Science, Department of Advanced Occupational Therapy, Department of Brain Function Rehabilitation, Kyoto University

Riku Takebuchi

Third-year Undergraduate Student, Human Health Sciences, Course of Advanced Rehabilitation Science, Department of Advanced Occupational Therapy, Faculty of Medicine, Kyoto University

Shiori Kinoshita

Third-year Undergraduate Student, Human Health Sciences, Course of Advanced Rehabilitation Science, Department of Advanced Occupational Therapy, Faculty of Medicine, Kyoto University

Yukinori Deguchi

Third-year Undergraduate Student, Human Health Sciences, Course of Advanced Rehabilitation Science, Department of Advanced Occupational Therapy, Faculty of Medicine, Kyoto University

3) Names, affiliations, positions, and roles of the sub-researchers

Role: Coordinates research and advises on planning, execution, analysis, and discussion.

Marie Furuta

Professor, Human Health Sciences, Course of Advanced Nursing Science, Division of Advanced Global Nursing Science, Perinatal Epidemiology, Graduate School of Medicine, Kyoto University

Chifumi Otaki

Lecturer, Human Health Sciences, Course of Advanced Nursing Science, Division of Advanced Global Nursing Science, Perinatal Epidemiology, Graduate School of Medicine, Kyoto University

Hitoshi Tanimukai

Associate Professor, Human Health Sciences, Graduate School of Medicine, Course of Advanced Rehabilitation Science, Department of Advanced Occupational Therapy, Department of Brain Function Rehabilitation, Kyoto University

Keisuke Irie

Lecturer, Occupational Therapist, Human Health Sciences, Graduate School of Medicine, Course of Advanced Rehabilitation Science, Department of Advanced Occupational Therapy, Department of Clinical Cognitive Neuroscience, Kyoto University

Taisuke Mori

Occupational Therapist, Day Care Service Department, Kyoto University Hospital

4) Name, affiliation, and position of the person responsible for the management of the sample/information

Hiroyuki Inadomi

Professor, Human Health Sciences, Graduate School of Medicine, Course of Advanced Rehabilitation Science, Department of Advanced Occupational Therapy, Department of Brain Function Rehabilitation, Kyoto University

5) Name, affiliation, and position of the person in charge of statistical analysis and data management

Marie Furuta

Professor, Human Health Sciences, Course of Advanced Nursing Science, Division of Advanced Global Nursing Science, Perinatal Epidemiology, Graduate School of Medicine, Kyoto University

Chifumi Otaki

Lecturer, Human Health Sciences, Course of Advanced Nursing Science, Division of Advanced Global Nursing Science, Perinatal Epidemiology, Graduate School of Medicine, Kyoto University

18. Changes and revisions to the research protocol

When changes or revisions to the research protocol are required, the researcher should apply to the Ethical Review Committee for approval once again.

19. Ethical guidelines to be followed

This research will be conducted in accordance with the "Declaration of Helsinki" and the "Ethical Guidelines for Life Sciences and Medical Research Involving Human Subjects".

20. Attribution of research results

The results of this research shall belong to Kyoto University.

21. References

1. Thye, M. D., Bednarz, H. M., Herringshaw, A. J., Sartin, E. B., & Kana, R. K. (2018). The impact of atypical sensory processing on social impairments in autism spectrum disorder. developmental cognitive neuroscience, 29, 151-167.
2. Green, M. F., Horan, W. P., & Lee, J. (2015). Social cognition in schizophrenia.Nature Reviews Neuroscience, 16(10), 620-631.
3. Sutton, D., Wilson, M., Van Kessel, K., & Vanderpyl, J. (2013). Optimizing arousal to manage aggression: A pilot study of sensory modulation. international journal of mental health nursing, 22(6), 500-511.
4. Burns, P., & Van Der Meer, R. (2021). Happy Hookers: findings from an international study exploring the effects of crochet on wellbeing. Perspectives in public health, 141(3), 149-157.
5. Shimada, T., Ohori, M., Inagaki, Y., Shimooka, Y., Sugimura, N., Ishihara, I., ... & Kobayashi, M. (2018). A multicenter, randomized controlled trial of individualized occupational therapy for patients with schizophrenia in Japan. PLoS One, 13(4), e 0193869.
6. DeBoth, K. K., Reynolds, S., Lane, S. J., Carretta, H., Lane, A. E., & Schaaf, R. C. (2021). Neurophysiological Correlates of Sensory-Based Phenotypes in ASD. Child Psychiatry & Human Development, 1-13.
7. Rissling, A. J., & Light, G. A. (2010). Neurophysiological measures of sensory registration, stimulus discrimination, and selection in schizophrenia patients. of schizophrenia and its treatment, 283-309.
8. Yasuma, F., & Hayano, J. I. (2004). Respiratory sinus arrhythmia: why does the heartbeat synchronize with respiratory rhythm? Chest, 125(2), 683-690.
9. Zlotnik, S., Attias, J., Pratt, H., & Engel-Yeger, B. (2018). Neurophysiological manifestations of auditory hypersensitivity correlate with daily life experiences. neuroscience and medicine, 9(01), 29.
10. van den Boogert, F., Spaan, P., Sizoo, B., Bouman, Y. H., Hoogendijk, W. J., & Roza, S. J. (2022). Sensory Processing, Perceived Stress and Burnout Symptoms in a Working Population during the COVID-19 Crisis. Research and Public Health, 19(4), 2043.
11. Sulik, M. J., Eisenberg, N., Spinrad, T. L., & Silva, K. M. (2015). Associations between respiratory sinus arrhythmia (RSA) reactivity and effortful control in preschool-aged children. psychobiology, 57(5), 596-606.
12. Tininenko, J. R., Measelle, J. R., Ablow, J. C., & High, R. (2012). Respiratory control when measuring respiratory sinus arrhythmia during a talking task. biological psychology, 89(3), 562-569.
13. Schaaf, R. C., Benevides, T. W., Blanche, E., Brett-Green, B. A., Burke, J., Cohn, E., ... & Schoen, S. A. (2010). Parasympathetic functions in children with sensory processing disorder. frontiers in integrative neuroscience, 4, 4.
14. Chalmers, A., Harrison, S., Mollison, K., Molloy, N., & Gray, K. (2012). Establishing sensory-based approaches in mental health inpatient care: a multidisciplinary approach. Australasian Psychiatry, 20(1), 35-39.
15. Wiglesworth, S., & Farnworth, L. (2016). An exploration of the use of a sensory room in a forensic mental health setting: staff and patient perspectives. *Occupational Therapy International*, *23*( 3), 255-264.
16. Koshiyama, D., Kirihara, K., Tada, M., Nagai, T., Fujioka, M., Usui, K., ... & Kasai, K. (2020). Reduced auditory mismatch negativity reflects impaired deviance detection in schizophrenia. schizophrenia bulletin, 46(4), 937-946.
