# Supplementary material for: Effects of sensory room intervention on autonomic function in healthy adults: A pilot randomized controlled trial
Source: PLoS One. 2025 Apr 23;20(4):e0319649. doi: 10.1371/journal.pone.0319649 (PMC12017487; doi:10.1371/journal.pone.0319649)
Supplement: S2 Table — (DOCX) [file pone.0319649.s005.docx]

**S2 Table. The mean values before and after the intervention for the two groups.**

|  | **SA, n=17^1^** | | **SRI, n=20^1^** | |
| --- | --- | --- | --- | --- |
| Variable | **Pre** | **Post** | **Pre** | **Post** |
| RSA (In[ms^2^]) |  |  |  |  |
| Resting | 6.4 (1.0) | 6.1 (1.0) | 6.0 (0.9) | 6.5 (1.0) |
| Tones | 6.5 (0.9) | 6.2 (0.8) | 6.3 (0.8) | 6.5 (1.0) |
| Visual | 6.1 (0.8) | 6.0 (0.8) | 6.1 (0.9) | 6.3 (1.0) |
| Siren | 6.3 (0.9) | 6.1 (0.7) | 6.2 (0.8) | 6.2 (0.8) |
| Olfactory | 6.6 (0.9) | 6.4 (0.7) | 6.4 (0.9) | 6.4 (0.7) |
| Tactile | 6.4 (0.8) | 6.2 (0.7) | 6.2 (0.8) | 6.3 (0.7) |
| Vestibular | 6.3 (0.8) | 6.4 (0.8) | 6.5 (1.0) | 6.7 (0.9) |
| Recovery | 6.1 (0.7) | 6.1 (0.7) | 6.1 (1.1) | 6.2 (0.9) |
| Prolonged auditory | 6.1 (0.6) | 6.0 (0.9) | 6.1 (1.0) | 6.2 (1.0) |
| POMS2 |  |  |  |  |
| Anger/Hostility | 45.8 (7.9) | 40.6 (4.8) | 42.9 (9.3) | 38.7 (1.8) |
| Confusion/Bewilderment | 51.1 (10.9) | 43.3 (6.9) | 50.0 (11.1) | 42.6 (7.4) |
| Depression/Dejection | 47.2 (7.1) | 42.8 (4.0) | 45.4 (4.9) | 42.2 (3.4) |
| Fatigue/Inertia | 47.6 (9.9) | 42.9 (8.3) | 44.9 (7.4) | 41.4 (7.3) |
| Tension/Anxiety | 50.5 (12.3) | 43.5 (7.9) | 49.0 (10.8) | 37.2 (5.9) |
| Vigor/Activity | 57.2 (10.1) | 60.6 (12.5) | 55.6 (9.4) | 55.7 (10.5) |
| Friendliness | 52.6 (11.1) | 45.8 (14.5) | 58.0 (10.2) | 50.5 (12.9) |
| TMD | 46.9 (9.7) | 40.2 (7.3) | 45.0 (8.1) | 39.0 (5.3) |
| CAB-AT |  |  |  |  |
| Updating | 433.4 (213.2) | 595.1 (177.4) | 491.4 (166.6) | 589.8 (171.4) |
| Inhibition | 606.2 (123.0) | 635.9 (84.7) | 553.8 (181.8) | 641.6 (76.6) |
| Divided attention | 618.4 (146.3) | 687.4 (117.1) | 608.5 (163.9) | 657.0 (143.1) |
| Focus attention | 761.2 (89.1) | 746.2 (97.2) | 741.2 (141.7) | 728.1 (139.2) |
| Total | 604.8 (81.3) | 666.0 (87.3) | 598.5 (103.8) | 654.2 (104.5) |
| ^1^Mean (SD) |  |  |  |  |

Note. SA, Sedentary activity; SRI, Sensory room intervention; RSA, Respiratory sinus arrhythmia; POMS2, Profile of Mood States 2nd Edition; TMD, Total mood disturbance; CAB-AT, Concentration Cognitive Assessment.
