# Supplementary material for: Effects of sensory room intervention on autonomic function in healthy adults: A pilot randomized controlled trial
Source: PLoS One. 2025 Apr 23;20(4):e0319649. doi: 10.1371/journal.pone.0319649 (PMC12017487; doi:10.1371/journal.pone.0319649)
Supplement: S3 Table — Values indicate the number of people using each item for each usage time. (DOCX) [file pone.0319649.s006.docx]

**S3 Table. Activities engaged in the Sedentary Activity group (n=17).**

| Items | 0 < t < 10 min | 10 ≤ t < 20 min | 20 ≤ t < 30 min | Total |
| --- | --- | --- | --- | --- |
| Crosswords | 2 | 1 | 0 | 3 |
| Sudoku | 0 | 4 | 4 | 8 |
| Jigsaw puzzles | 1 | 1 | 6 | 8 |
| Rubik's cubes | 1 | 2 | 0 | 3 |
| Origami | 2 | 0 | 2 | 4 |
| Coloring books | 1 | 0 | 0 | 1 |
| Macramé | 1 | 0 | 0 | 1 |
| Magazines | 1 | 0 | 0 | 1 |
| Comic books | 3 | 1 | 0 | 4 |

Note. Values indicate the number of people using each item for each usage time. t: usage time.

The participants were more likely to choose jigsaw puzzles (n=8) and Sudoku (n=8).
