# Supplementary material for: Effects of sensory room intervention on autonomic function in healthy adults: A pilot randomized controlled trial
Source: PLoS One. 2025 Apr 23;20(4):e0319649. doi: 10.1371/journal.pone.0319649 (PMC12017487; doi:10.1371/journal.pone.0319649)
Supplement: S4 Table — Values indicate the number of people using each item for each usage time. (DOCX) [file pone.0319649.s007.docx]

**S4 Table. Sensory items used in the Sensory Room Intervention group (n=20).**

| Items | 0 < t < 10 min | 10 ≤ t < 20 min | 20 ≤ t < 30 min | Total |
| --- | --- | --- | --- | --- |
| Life-size beaded cushion | 0 | 3 | 12 | 15 |
| Small beaded cushions | 0 | 1 | 16 | 17 |
| Balance ball | 0 | 1 | 3 | 4 |
| Stretch pole | 0 | 0 | 1 | 1 |
| Hugging pillow | 2 | 0 | 11 | 13 |
| Weighted blanket | 1 | 0 | 13 | 14 |
| Light blanket | 0 | 1 | 9 | 10 |
| Squeezes | 4 | 3 | 7 | 14 |
| Mini bubble tube | 0 | 0 | 18 | 18 |
| Rocking chair | 0 | 4 | 6 | 10 |
| Electric hand massager | 8 | 1 | 2 | 11 |
| Foot roller | 6 | 1 | 1 | 8 |
| Music player with healing music | 0 | 0 | 20 | 20 |
| Aroma diffuser | 0 | 0 | 20 | 20 |

Note. Values indicate the number of people using each item for each usage time. t: usage time.

All the participants listened to healing music, used one of the aroma oils, and leaned on either a life-size cushion or a rocking chair. All but two participants used bubble tubes. A balance ball was used as a footrest for rocking chairs by all users, and a foot roller was used for the upper limb. The most popular aroma oils were lemon (n=6), lavender (n=5), orange (n=4), eucalyptus (n=3), and tea tree (n=2).
