# Supplementary material for: Effects of sensory room intervention on autonomic function in healthy adults: A pilot randomized controlled trial
Source: PLoS One. 2025 Apr 23;20(4):e0319649. doi: 10.1371/journal.pone.0319649 (PMC12017487; doi:10.1371/journal.pone.0319649)
Supplement: S1 Fig — (DOCX) [file pone.0319649.s008.docx]

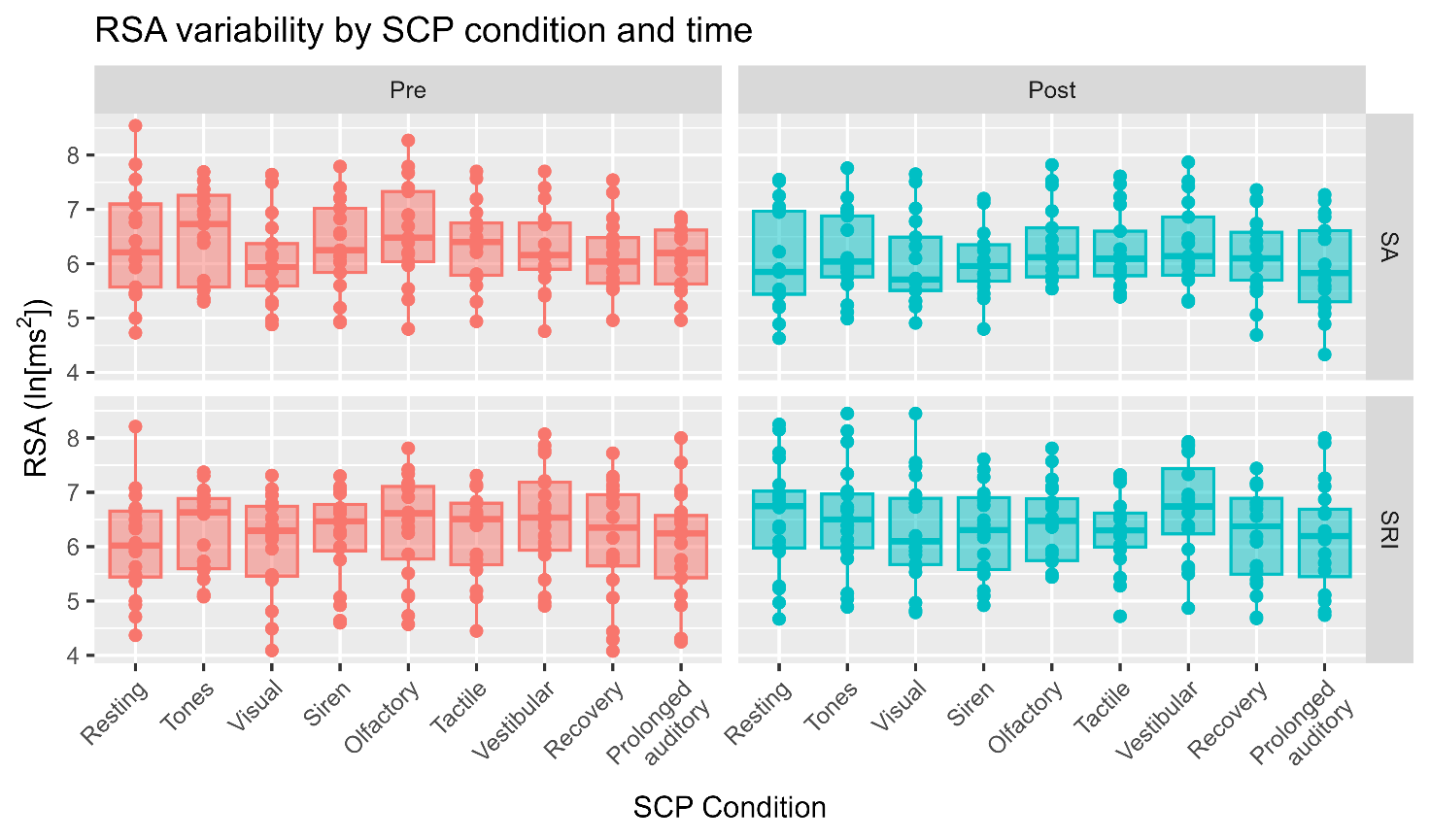


**S1 Fig. RSA between pre- and post-intervention during the SCP.**

Note. RSA, Respiratory sinus arrhythmia; SCP, Sensory challenge protocol; SA, Sedentary activity; SRI, Sensory room intervention.
