# Supplementary material for: Effects of sensory room intervention on autonomic function in healthy adults: A pilot randomized controlled trial
Source: PLoS One. 2025 Apr 23;20(4):e0319649. doi: 10.1371/journal.pone.0319649 (PMC12017487; doi:10.1371/journal.pone.0319649)
Supplement: S1 Appendix — (DOCX) [file pone.0319649.s009.docx]

**S1 Appendix. Details of the sensory room items**

| **Item** | **Manufacturer** |
| --- | --- |
| life-size beaded cushion | Yogibo Max, Yogibo, Japan |
| rocking chair | rocking chair with adjustable angles for the head and back, Taira Interior Industry, Japan |
| mini bubble tube | ROMPA, UK |
| two small beaded cushions | "Kumo ni Sawaru Yume Cushion," MOGU, Japan |
| balance ball | Core Balance Exercise Ball, NEEDS LABO, Japan |
| stretch pole | Repure Stress-Free Stretch Pillow (Pole Type), Tanaka Futon Service, Japan |
| hugging pillow | "Premium Kimochi Ii Dakimakura," MOGU, Japan |
| weighted blanket | MERRYLIFE, USA |
| light blanket | "bon moment" Blanket, Selecture, Japan |
| two squeezes | Sticky Stress Balls, JML Market Corp., USA |
| electric hand massager | Hand Massager MD-8301, THRIVE, Daito Electric, Japan |
| foot roller | Massage Roller, Rozally, Japan |
| healing music | "MENTAL DETOX for Inner Beauty and Energy," Della Inc., Japan |
| aroma oils (orange, lemon, lavender, eucalyptus, and tea tree) | Amazing Craft, Japan |
