# Supplementary material for: Effects of sensory room intervention on autonomic function in healthy adults: A pilot randomized controlled trial
Source: PLoS One. 2025 Apr 23;20(4):e0319649. doi: 10.1371/journal.pone.0319649 (PMC12017487; doi:10.1371/journal.pone.0319649)
Supplement: S2 Appendix — (DOCX) [file pone.0319649.s010.docx]

## **S2 Appendix.** Details of the Sensory Challenge Protocol.

The following table describes each stimulus in the Sensory Challenge Protocol (SCP). Each stimulus, except for the prolonged auditory task, lasted approximately 3 seconds with inter-stimulus intervals of 12–17 seconds and was repeated eight times. The video consistently showed cross marks, provided timing for all stimuli, and presented visual and auditory stimuli.

| **SCP condition** | **Duration, frequency** | **Contents** |
| --- | --- | --- |
| Resting | 3 min. | Looking at the fixation point on the display |
| Tones | 3s × 8 times | 84 dB and 1.7 Hz alarm tone |
| Visual | 3s × 8 times | 20 W and 10 Hz flashing light |
| Siren | 3s × 8 times | 78 dB fire siren |
| Olfactory | 3s × 8 times | The tester held wintergreen oil under the participant's nose. |
| Tactile | 3s × 8 times | The tester touched the participant's chin from left to right to left with a feather measuring approximately 7 cm. |
| Vestibular | 3s × 8 times | The tester stood behind the reclining chair on which the participant was sitting and tilted the back of the chair 30 degrees for 1 second, held this position for 1 second and returned it to its original position for 1 second. |
| Recovery | 3 min. | Looking at the fixation point on the display |
| Prolonged auditory | 2 min. | Continuous sound at 75 dB |
