# Supplementary material for: Effects of sensory room intervention on autonomic function in healthy adults: A pilot randomized controlled trial
Source: PLoS One. 2025 Apr 23;20(4):e0319649. doi: 10.1371/journal.pone.0319649 (PMC12017487; doi:10.1371/journal.pone.0319649)
Supplement: S3 Appendix — (DOCX) [file pone.0319649.s011.docx]

## **S3 Appendix.** Details of the statistical analysis.

We implemented the following code using brms package for Bayesian modeling to analyze value of respiratory sinus arrhythmia (RSA), Profile of Mood States 2nd Edition (POMS2) and Concentration Cognitive Assessment (CAB-AT).

*# package*

*library(brms)*

*# RSA analysis*

*brm(formula = RSA ~ Treatment*Time*Condition + (1|ID),*

*data = data,*

*chains = 6,*

*cores = 8,*

*iter = 8000,*

*warmup = 1000,*

*seed = 123,*

*family = gaussian,*

*save_pars = save_pars(all = TRUE)*

*)*

*# for POMS2 and CAB-AT analysis*

*brm(formula = value ~ Time*Treatment + (1|ID),*

*data = data,*

*chains = 6,*

*cores = 8,*

*iter = 8000,*

*warmup = 1000,*

*seed = 123,*

*family = gaussian,*

*save_pars = save_pars(all = TRUE)*

*)*
